# Metabolic switching, growth kinetics and cell yields in the scalable manufacture of stem cell-derived insulin-producing cells

**DOI:** 10.1186/s13287-023-03574-3

**Published:** 2024-01-02

**Authors:** Diepiriye G. Iworima, Robert K. Baker, Cara Ellis, Chris Sherwood, Lisa Zhan, Alireza Rezania, James M. Piret, Timothy J. Kieffer

**Affiliations:** 1 Department of Cellular and Physiological Sciences, Life Sciences Institute, The University of British Columbia, Vancouver, BC Canada; 2 School of Biomedical Engineering, The University of British Columbia, Vancouver, BC Canada; 3 Michael Smith Laboratories, The University of British Columbia, Vancouver, BC Canada; 4 CRISPR Therapeutics, Boston, MA USA; 5 Department of Chemical and Biological Engineering, The University of British Columbia, Vancouver, BC Canada; 6 Department of Surgery, The University of British Columbia, Vancouver, BC Canada

**Keywords:** Bioprocess development, Cell yield, Pluripotent stem cells, Diabetes, Beta cells, Islets

## Abstract

**Background:**

Diabetes is a disease affecting over 500 million people globally due to insulin insufficiency or insensitivity. For individuals with type 1 diabetes, pancreatic islet transplantation can help regulate their blood glucose levels. However, the scarcity of cadaveric donor islets limits the number of people that could receive this therapy. To address this issue, human pluripotent stem cells offer a potentially unlimited source for generating insulin-producing cells through directed differentiation. Several protocols have been developed to make stem cell-derived insulin-producing cells. However, there is a lack of knowledge regarding the bioprocess parameters associated with these differentiation protocols and how they can be utilized to increase the cell yield.

**Methods:**

We investigated various bioprocess parameters and quality target product profiles that may influence the differentiation pipeline using a seven-stage protocol in a scalable manner with CellSTACKs and vertical wheel bioreactors (PBS-Minis).

**Results:**

Cells maintained > 80% viability through all stages of differentiation and appropriately expressed stage-specific markers. During the initial four stages leading up to the development of pancreatic progenitors, there was an increase in cell numbers. Following pancreatic progenitor stage, there was a gradual decrease in the percentage of proliferative cells, as determined by Ki67 positivity, and a significant loss of cells during the period of endocrine differentiation. By minimizing the occurrence of aggregate fusion, we were able to enhance cell yield during the later stages of differentiation. We suggest that glucose utilization and lactate production are cell quality attributes that should be considered during the characterization of insulin-producing cells derived from stem cells. Our findings also revealed a gradual metabolic shift from glycolysis, during the initial four stages of pancreatic progenitor formation, to oxidative phosphorylation later on during endocrine differentiation. Furthermore, the resulting insulin-producing cells exhibited a response to several secretagogues, including high glucose.

**Conclusion:**

This study demonstrates process parameters such as glucose consumption and lactate production rates that may be used to facilitate the scalable manufacture of stem cell-derived insulin-producing cells.

**Supplementary Information:**

The online version contains supplementary material available at 10.1186/s13287-023-03574-3.

## Introduction

Type 1 diabetes (T1D) is a metabolic disorder caused by the autoimmune destruction of beta cells in the pancreatic islets of Langerhans, resulting in hyperglycemia [1]. As of 2021, there were an estimated 537 million people worldwide living with some form of diabetes, with T1D accounting for approximately 10% of reported cases [2]. While exogenous insulin injections are the most common therapy for managing the disease, they are not as effective as the pancreatic islets in maintaining glycemic control. Transplanting cadaveric donor islets is an effective cell replacement therapy that can lead to lower blood glucose levels and lower exogenous insulin requirements [3, 4]. However, the use of islet transplantation as therapy is limited by the scarcity of donor islets.

Human pluripotent stem cells (hPSCs), including embryonic and induced pluripotent stem cells (hESCs and iPSCs, respectively), are a renewable source of starting material for cell replacement therapy. Several protocols have been developed to generate hPSC-derived pancreatic progenitors and insulin-producing islet-like clusters capable of preventing or reversing chemically induced diabetes in rodents [5–10]. These stepwise protocols use similar strategies: (1) an exit from the pluripotent state followed by the induction of *SOX17*- and *FOXA2*-expressing definitive endoderm (DE), (2) induction of the primitive gut tube, (3) the generation of multipotent pancreatic progenitors expressing *PDX1* and *NKX6.1*, (4) *NGN3*-expressing endocrine progenitors and (5) maturing endocrine cells producing insulin. Achieving similar functionality and transcriptomic profiles to donor human islets remains challenging in most protocols. In addition, manufacturing clinically relevant numbers of cells is a bottleneck. Additionally, there are limited reports characterizing bioprocess parameters beyond typical biomarkers and functional assays, with even fewer studies reporting the cell yields associated with differentiation [11, 12, 13].

The goal of this study was to develop a differentiation protocol guided by the Quality-by-Design principles [14], where both the stem cell derivatives and process parameters were characterized. We report a seven-stage differentiation protocol to generate hPSC-derived insulin-producing cells using a monolayer-suspension hybrid workflow that is amenable to scaling-up. A quality target product profile (QTPP) is a component of Quality-by-Design that provides an overview of all the elements that impact quality, safety and efficacy of a desired product for clinical use [14]. Throughout the differentiation, we summarize the QTPP, including critical quality attributes (CQAs), physical, chemical, microbiological or biological properties that ensure the quality of the product and critical process parameters (CPPs), defined as process parameters that impact CQAs. We improved cell yields while maintaining high viability by controlling the aggregate size distribution using chemical and bioprocess modifications in the later differentiation stages. We found distinct nutrient utilization phenotypes suggesting a metabolic switch from glycolysis during the earlier stages, to oxidative phosphorylation (OXPHOS) during the later stages of the differentiation. With our protocol, we can make insulin-producing cells that secrete insulin following stimulation with several secretagogues. We propose that lactate production and glucose consumption rates be considered and incorporated as CQAs. Insights from this study may contribute to the knowledge base for larger-scale production of hPSC-derived pancreatic progenitors and insulin-producing cells.

## Materials and methods

### Cell sources and culture

H1 and H9 cells obtained from WiCell were used for experiments. Cells were cultured on a feeder-free monolayer using serum-free media mTeSR^TM^1 (STEMCELL Technologies, Cat# 85850). The cells were passaged as clumps using Gentle Cell Dissociation Reagent (GCDR) (STEMCELL Technologies, Cat #100–0485) when the confluence was ~ 80%. Cell clumps were reseeded on Matrigel-coated vessels in mTeSR^TM^1 supplemented with 10 µM Y27632 for the first 24 h (h). Daily complete media exchanges with mTeSR^TM^1 were done within ± 2 h of the previous fed time. Colony morphology was monitored daily and imaged using a Primovert microscope (ZEISS). All cells were differentiated within five passages post-thaw and were cultured under standard humidified conditions: 21% O_2_, 5% CO_2_, 37 °C.

Human islets were obtained from Alberta Diabetes Institute IsletCore with informed consent. Islet isolations were approved by the Human Research Ethics Board at the University of Alberta (Pro00013094). The use of human islets was approved by the University of British Columbia Clinical Research Ethics Board. Islets were cultured in CMRL 1066 supplemented, CIT modification media (Corning, Cat#98–304-CV), and the media were changed every other day. All donor islets used for this study are given in Table 1.

**Table 1:** Donor human islet used for the study

Donor ID	Age (yrs)	Sex	BMI	Prep purity (%)
r225	69	M	22.7	90
r226	30	F	32.2	95
r229	22	F	23	95
r234	50	F	31.7	90
r237	61	M	19.6	95
r271	60	F	26	95
r282	57	M	26.4	90
r290	74	F	35.8	80
r303	56	F	24.1	85
r319	69	M	27.8	90
r326	26	M	27	90
r356	45	F	29.7	80
r421	60	F	25.9	95
r434	59	F	24.2	85
r455	62	M	30.1	95

### Differentiation

**Fig. 1:**
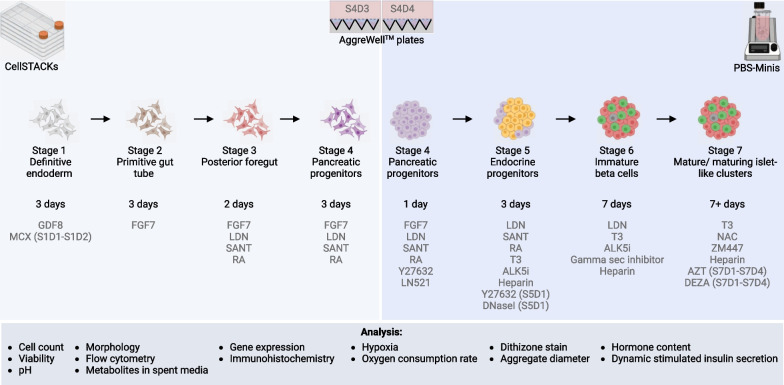
Schematic of the differentiation protocol workflow and assays performed. Stages 1–4 are performed on a 2D monolayer, stage 4 aggregates are made using AggreWell™ plates and stages 5–7 aggregates are cultured in vertical wheel PBS-Mini bioreactors. S denotes the stage and D denotes the day, e.g., S1D1 = stage 1 day 1

Confluent H1 cells were passaged as single cells with TrypLE™ Express (Invitrogen, Cat# 12604021) and reseeded at 90,000 cells/cm^2^ in mTeSR^TM^1 supplemented with 10 µM Y27632. Twenty-four hours later, the media were replaced with fresh unsupplemented mTeSR^TM^1. The differentiation was started ~ 48 h after the initial seeding using a combination of a modified version of our previously reported protocol [5] and patent US10633635B2 outlined in Table 2 and Fig. 1.

**Table 2:** Seven-stage differentiation protocol

Time line	Lineage	Basal media*		Growth factors	Small molecules
		Base	Supplement		
S0D0	Pluripotent stem cell	mTeSR^TM^1			10 µM Y27632
S0D1		mTeSR^TM^1			
S1D1–D3	Definitive endoderm	MCDB131	2 mM GlutaMAX	100 ng/ml GDF8	1 µM MCX (S1D1 only)
			0.5% FAF-BSA		0.1 µM MCX (S1D2 only)
			10 mM Glucose (Final concentration)		
			2.7 g/L bicarbonate (Final concentration)		
			1:5000 ITS-X (3D differentiation only)		
S2D1–D3	Primitive gut tube	MCDB131	2 mM GlutaMAX	50 ng/ml FGF7	
			1% FAF-BSA		
			10 mM Glucose (Final concentration)		
			2.7 g/L bicarbonate (Final concentration)		
			0.25 mM Vitamin C		
S3D1–D2	Posterior foregut	BLAR	2 mM GlutaMAX	25 ng/ml FGF7	1 µM Retinoic Acid
			2% FAF-BSA		0.25 µM SANT-1
			10 mM Glucose (Final concentration)		300 nM TPPB
			3.6 g/L bicarbonate (Final concentration)		250 nM LDN
			1:200 ITS-X		
			0.25 mM Vitamin C		
S4D1–D3	Pancreatic progenitor	BLAR	2 mM GlutaMAX	2 ng/ml FGF7	0.05 µM Retinoic Acid
			2% FAF-BSA		0.25 µM SANT-1
			10 mM Glucose (Final concentration)		200 nM TPPB
			3.6 g/L bicarbonate (Final concentration)		250 nM LDN
			1:200 ITS-X		
			0.25 mM Vitamin C		
S4D4 Aggrewell aggregation	Pancreatic progenitor	BLAR	2 mM GlutaMAX	2 ng/ml FGF7	0.05 µM Retinoic Acid
			2% FAF-BSA		0.25 µM SANT-1
			10 mM Glucose (Final concentration)		200 nM TPPB
			3.6 g/L bicarbonate (Final concentration)		250 nM LDN
			1:200 ITS-X		1:50 Laminin 521 (100 µg/mL stock)
			0.25 mM Vitamin C		10 µM Y27632
S5D1–D3 PBS-Mini: 60 rpm	Endocrine progenitor	BLAR	2 mM GlutaMAX		50 nM Retinoic Acid
			2% FAF-BSA		0.25 µM SANT-1
			20 mM Glucose (Final concentration)		10 µM ALK5i
			2.7 g/L bicarbonate (Final concentration)		100 nM LDN
			1:200 ITS-X		1 µM T3
			10 µM ZnSO4		10 µg/ml heparin
					10 µM Y27632 (S5D1 only)
					8 U/mL DNAse (S5D1 only)
S6D1-D7 PBS-Mini: 60 rpm	Immature endocrine cells	BLAR	2 mM GlutaMAX		10 µM ALK5i
			2% FAF-BSA		100 nM LDN
			20 mM Glucose (Final concentration)		1 µM T3
			2.7 g/L bicarbonate (Final concentration)		100 nM Gamma secretase inhibitor
			1:200 ITS-X		10 µg/mL heparin
			10 µM ZnSO4		
S7 D1–D8+ PBS-Mini: 60 rpm	Maturing endocrine islet-like cells	BLAR	2 mM GlutaMAX		10 nM T3
			2% FAF-BSA		1 mM N-Acetyl cysteine
			20 mM Glucose (Final concentration)		0.5 µM ZM 447
			1:200 ITS-X		10 µg/mL heparin
			1:200 RPMI Vitamin supplement		5 µM AZT (S7D1-D4 only)
			1:200 MEM Non-essential amino acid		1 µM DEZA (S7D1-D4 only)
			1:2000 Lipid concentrate		
			1:200 Sodium pyruvate		
			1:2000 Trace elements A		
			1:2000 Trace elements B		
			2.7 g/L bicarbonate (Final concentration)		

*Protect from light

**Fig. 2:**
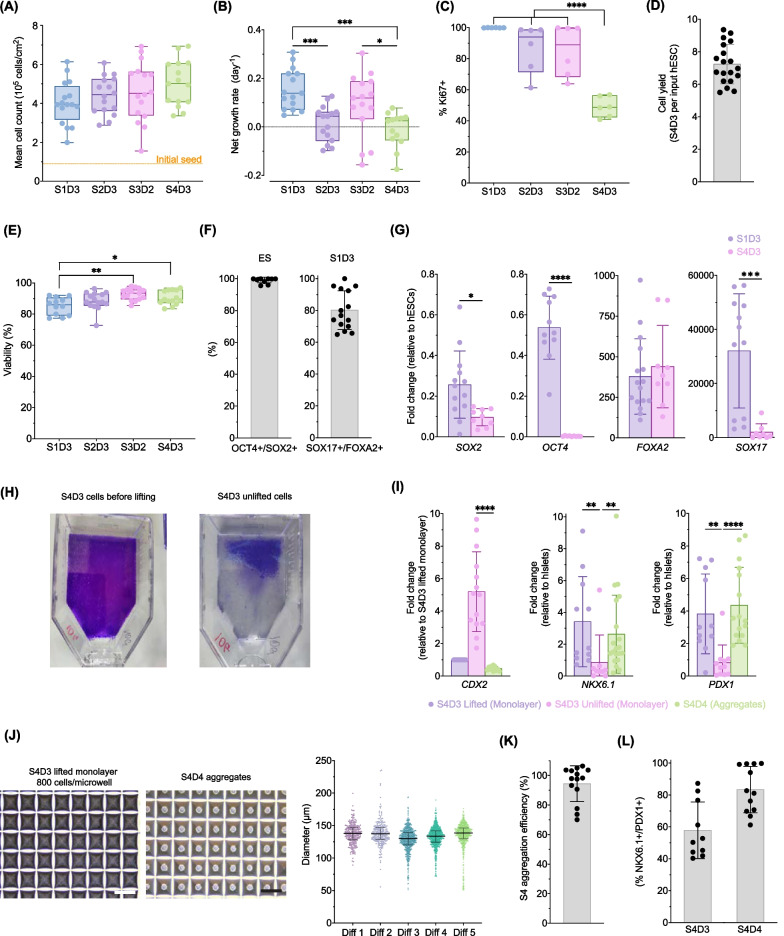
Characterization of stage-specific growth kinetics, viability and markers during the first four stages of differentiation. **A** Cell density during four-stage differentiation (*n* = 15–16 biological replicates, each biological replicate had 2 technical replicates; one-way ANOVA with Tukey post-test; orange dash line = initial seeding density). **B** Quantification of stage-specific net growth rate (*n* = 13–15 biological replicates; one-way ANOVA with Tukey post-test). **C** Ki67 positive cells using flow cytometry (*n* = 6 biological replicates; Brown–Forsythe and Welch ANOVA with Dunnett’s T3 post-test). **D** S4D3 cell yield per input hESC (*n* = 19 biological replicates). **E** Viability of cells during four-stage differentiation (*n* = 12–14 biological replicates; one-way ANOVA with Tukey post-test). **F** Quantification of stage-specific markers using flow cytometry (*n* = 9–15 biological replicates). **G** Gene expression profile of stage-specific markers relative to hESCs (*n* = 8–16 biological replicates; unpaired t-test) **H** Illustration of the selective lifting for *CDX2*^low^ population using crystal violet fixation of adherent cells at the end of S4D3. **I** Gene expression of *CDX2, NKX6.1* and *PDX1* in S4D3 lifted cells, S4D3 unlifted cells and S4D4 cell aggregates (*n* = 9–20 biological replicates; CDX2: two-tailed paired t-test; *NKX6.1* and *PDX1*: Brown–Forsythe and Welch ANOVA with Dunnett’s T3 post-test). **J** Representative images of S4D3 cells before and after overnight aggregation in the AggreWell™ plate, and aggregate diameter distribution from five independent differentiations, scale bar = 500 µm (*n* = 244–784 aggregates/group; plot shows individual aggregates with median and interquartile range). **K** Aggregation efficiency of S4D4 cells (*n* = 14 biological replicates). **L** Flow cytometry quantification of NKX6.1 + /PDX1 + positive cells at S4D3 and S4D4 (*n* = 10–12 biological replicates). All box and whisker plots show individual points with median and interquartile range; bar charts show individual points with mean ± SD. *P < 0.05, **P < 0.01, ***P < 0.001, ****P < 0.0001. S1D3 = stage 1 day 3, S2D3 = stage 2 day 3, S3D2 = stage 3 day 2, S4D3 = stage 4 day 3, S4D4 = stage 4 day 4

After differentiating the cells on a monolayer up to S4D3, single cells were harvested using TrypLE™ Express. First, the S4D3 cells were rinsed with PBS−/− and then incubated in TrypLE™ Express for 2 min at room temperature. Each vessel was lightly tapped for an additional 2 min to harvest the lifted cells before adding an equal volume of media to dilute the dissociation reagent. This cell harvesting method promotes the lifting of *CDX2*^low^ cells (Fig. 2I), while the remaining unlifted cells were collected for analysis using cell scrapers. The lifted cell suspension was spun at 1000 rpm for 5 min, and the supernatant was discarded. Cells were resuspended in S4D4 aggregation media, counted and reseeded at 800 cells/microwell in AggreWell™ plates (STEMCELL Technologies, Cat# 34425) following the manufacturer’s instructions. The cells were incubated for ~ 24 h, and the formed S4D4 aggregates were harvested as per the manufacturer's instructions and counted to determine the aggregation efficiency using the following equation:

$${\text{Aggregation}}\;{\text{efficiency}}\left( \% \right) = N_{f} /N_{i} \times 100$$

where *N*_*i*_ is the initial total cell count at S4D3 and *N*_*f*_ is the total cell count at S4D4. An aggregation efficiency of 100% suggests that all the initial single cells seeded were incorporated into the aggregates. Aggregates were reseeded into vertical wheel bioreactors (0.1 PBS-Minis) at ~ 0.5 to 1.4 × 10^6^ cells/mL, mixing at 60 rpm. Daily media changes were done between stages 1 and 6. Before starting S7D1, cell aggregates from two 0.1 PBS-Mini were combined into one vessel for further differentiation. Media were replaced every other day during stage 7.

For a four-stage differentiation starting with H1 PSC aggregates, harvested single cells were seeded into 0.1 PBS-Minis at a concentration of 1 million cells/mL mTeSR^TM^1 plus 10 µM Y27632 mixing at 60 rpm. Twenty-four hours after aggregate formation, the agitation was reduced to 40 rpm, and the media were replaced with mTeSR^TM^1 only. Forty-eight hours later, the aggregates were counted and reseeded at an appropriate cell concentration (0.2 − 1 × 10^6^ cells/mL), and the differentiation was initiated by mixing at 45 rpm following the protocol outlined in Table 2. To change the media while differentiating in the PBS-Mini, aggregates were collected in 50 mL Falcon tubes and spun gently at 800 rpm for 2 min, the supernatant was aspirated, and fresh media were gently used to resuspend aggregates before transferring them back to the bioreactor. All media changes regardless of the cell culture platform used (2D vs. 3D) were done within ± 2 h of the previous fed time.

When differentiating using the STEMdiff™ pancreatic progenitor kit (STEMCELL Technologies, Cat# 05120), a single cell suspension of H1 or H9 cells was initially seeded at 2e6 cells/well in six-well plates using mTeSR^TM^1 Plus (STEMCELL Technologies, Cat# 100–0276), supplemented with 10 µM Y27632. Twenty-four hours later, the differentiation was initiated according to the manufacturer’s instructions.

### Cell count, viability and hypoxia

To determine the growth kinetics of the cells during the differentiation, two T25 vessels (S1D3 -S4D3) or the contents of each bioreactor (S4D4–S7D8+) were evaluated at the end of each stage. At least duplicate counts were performed. Cell count and viability were obtained with a NC-200™, which uses acridine orange and 4’,6-diamidino-2-phenylindole (DAPI). The net growth rates and the doubling times were determined using the following equation:

$${\text{Net}}\;{\text{growth}}\;{\text{rate}}\;\left( {\mu_{{{\text{net}}}} } \right) = {\text{In}}\left( {N_{f} } \right){-}{\text{In}}\left( {N_{i} } \right)/{\triangle t}$$

$${\text{Doubling}}\;{\text{time}}\;\left( {T_{d} } \right) = In\left( 2 \right)/\mu_{{{\text{net}}}}$$

where *N*_*i*_ and *N*_*f*_ are the total cell count immediately before and at the end of a given stage, respectively, and Δt is the time elapsed at that stage.

Viability was also assessed using the LIVE/DEAD™ dyes (ThermoFisher Scientific, Cat# L3224). Briefly, aggregates were transferred to 24-well plates and incubated in 2 µM calcein-AM and 4 µM ethidium bromide, prepared in PBS+/+, for 30 min at room temperature in the dark. For the negative controls, cells were incubated in either 70% ethanol or dimethyl sulfoxide (DMSO) for 15 min at room temperature before staining with LIVE/DEAD™ dyes. Fluorescent images were captured with a AxioZoom V16 microscope (ZEISS).

Hypoxia stains were done using Image-iT™ Green Hypoxia Reagent (Invitrogen, Cat# I14834). The reagent was reconstituted as per the manufacturer’s instructions. Aggregates were stained with 5 µM Image-iT™ Green for 3 h under standard humidified conditions: 21% O_2_, 5% CO_2_, 37 °C.

### Morphology and aggregate size measurement

Monolayer and aggregate morphology was monitored daily via imaging using a Primovert microscope (ZEISS). Aggregates were randomly sampled into 6 well plates for the diameter measurements as previously described [15]. Three to six independent images were acquired for a total of 150–1500 aggregates. Aggregate diameters were measured using a semi-automatic pipeline in FIJI (ImageJ, NIH)**.** The background in each image was removed in Adobe Photoshop V19 or V20 (San Jose, USA) before further processing in FIJI. The masked images were visually compared to the original images before proceeding with the analysis. The area of each aggregate was determined, and the diameter was calculated using this equation:

$${\text{Diameter}}\;\left( d \right) = 2\surd (A/\pi),\;{\text{where}}\;A\;{\text{is}}\;{\text{the}}\;{\text{area}}\;{\text{of}}\;{\text{a}}\;{\text{given}}\;{\text{aggregate}}$$

### Flow cytometry

Cell monolayers or aggregates were dispersed as single cells using TrypLE™ Express (Invitrogen, Cat# 12604021) as outlined previously. After rinsing with PBS−/−, cell clusters were transferred to 6 well plates and incubated in TrypLE™ Express for up to 20 min at 37 °C. Every 5 min, the extent of aggregate dispersal was visually inspected, and clusters were gently triturated with a p1000 pipette. Once most aggregates were dispersed, double the volume of mTeSR^TM^1 was added to dilute TrypLE™ Express, and the cell suspension was passed through a 37 µm strainer. The single cells were spun (5 min, 1000 rpm), resuspended in PBS−/−, and cell count and viability were determined with a NC-200™. Cells were stained for viability with LIVE/DEAD™ fixable aqua dead cell stain (Invitrogen, Cat# L34957) using 0.5 µL stain/million cells for 30 min at room temperature in the dark. The cells were spun, rinsed in PBS −/− and resuspended in BD Cytofix/Cytoperm™ solution (BD Biosciences, Cat# 554714) for 30–60 min at room temperature or overnight at 4 °C. The fixed samples were then rinsed and resuspended in BD perm/wash™ solution (BD Biosciences, Cat# 554714) before performing the respective antibody stain (Table 3). Samples were incubated for 20 min at room temperature or overnight at 4 °C. All necessary antibody dilutions were done in BD Cytofix/CytoPerm™. Flow cytometry was performed using the LSRII-561, and the data were analyzed using FlowJo™ v10 software (BD Life Sciences).

**Table 3:** Flow cytometry antibodies

Antibody	Cat #	Dilution
SOX2-PE Mouse IgG2A; Clone 245,610	IC2018P	1000X
Alexa Fluor® 647 Mouse anti-Oct-3/4 40/Oct-3 RUO	560329	50X
Goat anti-human HNF-3 beta /FoxA2 Alexa Fluor® 488-conjugated antibody	IC2400G	100X
PE Mouse anti-Human Sox17 P7-969 RUO	561591	500X
Nkx6.1 APC	563338	50X
PE Mouse anti-PDX-1	562161	100X
Alexa Fluor® 647 Mouse Anti-C-Peptide U8-424 RUO	565831	2500X
PE Mouse Anti-Glucagon U16-850 RUO	565860	2500X
*Isotype controls*		
Ms IgG1 Kpa ItCl PE MOPC-21 100ug	554680	
Mouse IgG2A PE-conjugated Antibody	IC003P	
Ms IgG1Kpa ItCl Alexa 647 MOPC-21 isotype control	557732	
Normal Rabbit IgG Alexa Fluor® 488-conjugated Control	IC105G	
Ms IgG1Kpa ItCl Alexa 647 MOPC-21 100Tst	557714	
PE Mouse IgG1, κ Isotype Control	555749	

### Gene expression data

RNA extraction, cDNA synthesis and qPCR were done as previously described [16]. The data were normalized to undifferentiated H1 cells, human islets or S4D3 cells. The primer sequences used are given in Table 4.

**Table 4:** Primers used for the study

Primer	Forward	Reverse
SOX2	5′-GAGGAGAGTAAGAAACAGCATGGA-3′	5′-GATTGGTGTTCTCTTTTGCAGC-3′
NFX1	5′-TTTCAGAACAAAGGAGCTTCCAT-3′	5′-TTATCCACACAGCATATCTCATTACA-3′
OCT4	5′-GGGATTAAGTTCTTCATTCACTAAGGAA-3′	5′-CAAGAGCATCATTGAACTTCACCT-3′
FOXA2	5′-ATCGAGGACAAGTGAGAGAGCAA-3′	5′-TGTTATGGATTTCTTCTCCCTTGCG-3′
SOX17	5′-GGTATATTACTGCAACTATCCTGACG-3′	5′-GGAGTCTGAGGATTTCCTTAGCT -3′
CDX2	5′- GAGTTTCACTACAGTCGCTACATCA-3′	5′-GCTGCAACTTCTTCTTGTTGATTTTC -3′
NKX6.1	5′-CCTGTACCCCTCATCAAGGAT -3′	5′-CAAGTATTTTGTTTGTTCGAAAGTCTTC-3′
PDX1	5′-CCCTCTTTTAGTGATACTGGATTGG-3′	5′-CCTTCCAATGTGTATGGTACAGTTTC-3′
NGN3	5′-ACCACCCCATAATCTCATTCAAAG-3′	5′-GTAAGAGACTGAGAGGCAGACAG-3′
NEUROD1	5′- GGTTATGAGACTATCACTGCTCAG-3′	5′-AGAACTGAGACACTCGTCTGTC-3′
PAX4	5′-AGAGGCACTGGAGAAAGAGTTC -3′	5′-CCATTTGGCTCTTCTGTTGGA-3′
ARX	5′-CTCAGCACCACTCAAGACCAA -3′	5′-GCATCCAGACTGCTGTGAAG-3′
INS	5′-GCAGCCTTTGTGAACCAACA -3′	5′-GGTGTGTAGAAGAAGCCTCGTT-3′
GCG	5′-TTCTACAGCACACTACCAGAAGA -3′	5′-CTGGGAAGCTGAGAATGATCTG-3′
SST	5′-TCCGTCAGTTTCTGCAGAAGTC -3′	5′-CTGGGACAGATCTTCAGGTTCC-3′
MAFB	5′-GACTCCTGGCTTTCTGAACTTTG -3′	5′-CTCTCCTTTCCTCGTTGCTCTC-3
MAFA	5′-GTGAGTCCTGTGCTCAGTCG -3′	5′CTCTTGAAGGTTAAAACAAGATGTATTTCC3′
UNC3	5′-GATCAGTCAGTTTTACAGGTTGCT -3′	5′-GAAGGTTGGACAATACTGCACTTC-3′

### Immunohistochemistry

Aggregates were fixed in 4% paraformaldehyde (PFA) overnight, rinsed twice in PBS and embedded in 1% agarose. Embedded aggregates were fixed overnight in 4% PFA, resuspended in 70% ethanol and sectioned (Wax-it histology services, Vancouver, Canada). Slides were deparaffinized and hydrated using xylenes and graded alcohol series. Antigen retrieval was performed using a 10 mM sodium citrate buffer with 0.05% Tween 20 (pH 6). Slides were blocked for 10 min at room temperature in serum-free protein block (Dako, Cat# X0909) and then stained with primary antibodies diluted in antibody diluent (Dako, Cat# S3022) overnight at 4 °C. Primary antibody details are given in Table 5. Slides were rinsed thrice in PBS for 10 min and then stained with the appropriate secondary antibody for 1 h at room temperature. Following three more rinses in PBS, slides were counterstained with VECTASHIELD® HardSet™ mounting medium (Vector labs, Cat# H-1500). Slide scanning was done with a ImageXpressMicro™ and analyzed with MetaXpress software (Molecular Devices Corporation, Sunnyvale, CA).

**Table 5:** Immunohistochemistry antibodies

Antibody details			Dilution
Insulin	Thermo	PA1-26938	1:100
C-peptide	Abcam	Ab30477	1:100
Glucagon	Sigma	G 2654	1:1000
Somatostatin	Sigma	HPA019472	1:500
Trypsin	R&D	AF3586	1:25
Synaptophysin	Novus	NB120-16659	1:50
Cytokeratin 19	Dako	M0888	1:100
Insulin	Cell signaling	3014BF	1:200

### Crystal violet staining

To demonstrate our S4D3 selective lifting method, the monolayer was fixed, before or after cell harvest, in 2.3% crystal violet with 1% PFA and 1% methanol. Briefly, media were aspirated, and the T-flask was incubated in crystal violet solution for 20 min at room temperature on an orbital shaker (60 rpm). The fixative solution was decanted, and vessels were rinsed in water until all sediments were removed. Culture vessels were left to air dry, and images were captured using a OnePlus 9 Pro phone.

### Oxygen consumption rate

Oxygen consumption rate (OCR) was measured using a Resipher device (Lucid Scientific) or a Seahorse XFe96 analyzer (Agilent). For the stages done on a monolayer, single cells were harvested, counted and reseeded in 96-well plates for the Resipher device in stage-specific media plus 10 µM Y27632. Seeded plates were left at room temperature for 15 min before transferring to the incubator. Controls were run using undifferentiated H1 cells and MIN6 cells. For stages cultured in suspension, approximately 15 aggregates or human islets were placed in 96-well plates for the Resipher device. Live oxygen concentration was measured in real time for 17–23 h.

OCR and extracellular acidification rate (ECAR) were measured using a Seahorse XFe96 analyzer. Single cells were reseeded as described above and cultured overnight before testing. For the “mito stress test,” cells were incubated in a non-CO_2_ incubator for 1 h in serum-free Seahorse XF Base minimal DMEM media (Cat # 102353–100) supplemented with 10 mM glucose, 1 mM sodium pyruvate and 2 mM L-glutamine. Following measurement of basal respiration, the cells were treated with sequential injections of 1.5 µM oligomycin, 0.5 µM carbonyl cyanide-4-(trifluoromethoxy) phenyl hydrazone (FCCP) and 0.5 µM rotenone/antimycin A. OCR and ECAR were normalized to cell count.

### Dithizone staining

Dithizone (DTZ) (Sigma, Cat# 194832) powder was reconstituted in DMSO, diluted in PBS−/− and sterile-filtered to eliminate sediments. Clusters were stained in 5 mg/mL DTZ for ~ 2 min and rinsed with PBS−/−. Images were acquired using an AxioZoom V16 microscope (ZEISS).

### Human C-peptide content

S6 and S7 cells were suspended in cold lysis buffer containing 100 mM Tris–HCl, 300 mM NaCl and 2% nonidet P-40 and sonicated using a TissueLyser II (QIAGEN, Cat #85300) for 3 min (frequency = 300 s^−1^). The cell suspension was centrifuged (1 min, 1000 rcf, 4 °C) to remove cell debris. Cell lysates were stored at -80 °C until being assayed using the human C-peptide ELISA kit (ALPCO, Cat# 80-CPTHU-CH01). Hormone content was normalized to cell count.

### Dynamic perifusion

For the dynamic perifusion assay, approximately 15–20 stem cell-derived clusters or human islets were collected in a chamber. Assays were performed with two technical replicates. A modified triggering and amplifying protocol for insulin secretion was performed based on [17] (Additional file 6: Fig S6B). Briefly, clusters were equilibrated in 3 mM glucose Krebs–Ringer bicarbonate buffer (KRB; pH 7.4) (Table 6) at 100 µL/min for 32–48 min at 37 °C, and then, perifusate fractions were collected for analysis. Each secretagogue exposure was for 16 min, except the final exposure to low glucose for 40 min. Clusters were exposed to 3 mM glucose (3G) and then challenged with 16.7 mM glucose (16.7G), and then, 16.7G plus10 nM exendin-4, then 100 µM diazoxide, 100 µM tolbutamide and 30 µM forskolin were added in a stepwise manner. Effluent fractions were collected every 2 min. Afterward, clusters were lysed in cold lysis buffer containing 100 mM Tris–HCl, 300 mM NaCl and 2% nonidet P-40. Human insulin was quantified using radioimmunoassay (Millipore, Human Insulin RIA H1-14 K).

**Table 6:** KRB buffer composition

Chemical	mM (OR %)
NaCl	129.0
KCl	4.8
CaCl_2_(2H_2_O) dihydrate	2.5
MgSO_4_	1.2
Na_2_HPO_4_(2H_2_O)	1.0
KH_2_PO_4_	1.2
NaHCO_3_	5.0
Glucose	3.0
HEPES	1%
BSA	0.40%

### Metabolite assessment of spent media

Spent media collected from several time points were centrifuged to remove cell debris and frozen at −80 °C until being assayed. Before freezing, the pH was measured (Fisher Scientific, Cat# 13–636-AB15). Glucose and lactate were measured using a Stat Profile pHOx Ultra blood gas analyzer (Nova Biomedical). Nutrient consumption or metabolite production rates were calculated using the following equation:

$$Q_{{{\text{glu}}}} {\text{or }}Q_{{{\text{lac}}}} = S_{f} {-} \, S_{0} /\left( {\left[ {N/V_{f} } \right] \, \times \ \triangle t} \right)$$

where *Q*_glu_ or *Q*_lac_ refers to glucose consumption or lactate production rate, respectively. *S*_*f*_ and *S*_0_ are the concentrations of glucose or lactate in the spent and fresh media, respectively. *N* and *V*_*f*_ refer to the cell number and the media volume at the time of collection, respectively. Δ*t* is the time between media change.

Amino acid concentrations were measured using high-performance liquid chromatography. Hormone levels were measured using the following ELISA kits: human proinsulin (Mercodia, Cat# 10-1118-01) and human C-peptide (ALPCO, Cat #80-CPTHU-CH01).

### Statistical analysis

Statistical analysis was performed with GraphPad Prism (statistical tests used are specified in each figure legend). Data are shown as either an interquartile range with max and min values or mean ± standard deviation (SD).

## Results

### Characterization of stage-specific growth kinetics, viability and markers during the first four stages of differentiation

We previously described a differentiation protocol that generates insulin-producing cells capable of reversing STZ-induced diabetes in mice [5]. During the first four stages of differentiation toward pancreatic progenitors, off-target *CDX2*-expressing cells may arise, impacting the purity of the PDX1+ NKX6.1+ population [18]. *CDX2* expression is upregulated through BMP signaling [19, 20]. In this study, we made modifications to a previously published differentiation protocol [5] by: (1) prolonging the time in stage 2 media from 2 to 3 days, (2) increasing the concentration of the BMP inhibitor LDN during stage 4 and (3) introducing a cell selection step that targets CDX2^low^-expressing cells before stage 4 aggregation. Our previous method of generating pancreatic progenitor aggregates using air–liquid interface [5] was replaced with AggreWell™ plates or PBS-Mini bioreactors due to limitations in scalability.

We characterized the morphology, growth kinetics, aggregate formation and key markers during the first four stages of the differentiation (Fig. 2 and Additional file 1: Fig. S1). The cell density and number steadily increased between S1D3 and S4D3 (Fig. 2A and Additional file 1: Fig. S1A). The stage-specific net growth rates were, on average, positive, with the fastest growth rates at S1D3 and S3D2 (0.16 d^−1^ and 0.09 d^−1^, respectively) (Fig. 2B). The stage-specific doubling times were between 4 and 6 days (range: 2–27 days) (Additional file 1: Fig. S1B). Furthermore, there was an increase in the cell confluence, suggesting higher growth rates compared to death rates (Additional file 1: Fig. S1A). Notably, we observed substantial amounts of detached cells during media exchanges after S1D1 and S1D2. These presumed dead cells did not have a negative impact on the differentiation and this phenomenon dramatically decreased thereafter. In the event of substantial cell losses leading to reduced cell packing and confluence beyond stage 1, the cells would eventually be unable to differentiate into pancreatic progenitors, and the cultures were promptly terminated. Over time, the number of proliferative cells, as determined by Ki67+ staining, steadily decreased (Fig. 1C). On average, approximately seven S4D3 cells were produced per input human embryonic stem cell (Fig. 2D), while maintaining cell viability above 80% (Fig. 2E).

We evaluated the cells before the start of each differentiation and at the end of S1D3. Both the cell viability and OCT4+/SOX2+ population were ≥ 90%, indicating that we used high-quality hPSCs for the differentiations (Fig. 2F and Additional file 1: Fig. S1C-D). By S1D3, DE was successfully induced, as indicated by a population of ~ 80% FOXA2+/SOX17+ cells (Fig. 2F and Additional file 1: Fig. S1D). While *FOXA2* remained upregulated, *SOX17*, *SOX2* and *OCT4* expression dropped significantly by S4D3 (Fig. 2G). CDX2 is a marker of off-target cell types that may arise, including intestinal cells from the anterior foregut [21] and enterochromaffin-like cells [22, 23]. With this in mind, we developed a simple method to harvest *CDX2*^low^-expressing cells by incubating the monolayer at room temperature, followed by gentle tapping. Most S4D3 cells lifted, as indicated by the remaining crystal violet-fixed cells (Fig. 2H). The S4D3 cells that remained attached had higher *CDX2* and lower *NKX6.1* and *PDX1* than the lifted fraction of S4D3 cells (Fig. 2I). The lifted S4D3 cells were > 90% viable (Additional file 1: Fig. S1E) and S4D4 aggregates were made by seeding 800 single cells per microwell and culturing overnight in the presence of 10 µM Y27632 and laminin-521, which promote cell survival [24] and provide supportive extracellular matrix proteins [25, 26]. This aggregation process was robust and reproducible. Aggregates were relatively symmetrical spheres with a smooth periphery and a median diameter of ~ 130 µm (Fig. 2J). We recovered > 90% of the cells seeded (Fig. 2K). In addition to the upregulation of *NKX6.1* and *PDX1*, the mean NKX6.1+/PDX1+ population increased from ~ 60% at S4D3 to 80% (range 60–99%) by S4D4 (Fig. 2L and Additional file 1: Fig. S1F).

Given the limited scalability of this aggregation method, we also generated pancreatic progenitors using PBS-Minis. S4D3 single cells were seeded into 0.1 PBS-Minis with S4D4 media containing 8 U/mL DNase I mixing at 60 rpm. There was no difference in aggregation efficiency between PBS-Minis and AggreWell™ plates (*P* = 0.19) (Additional file 1: Fig. S1G). Furthermore, S4D4 aggregates were relatively homogenous in size (median ~ 85 µm) regardless of the initial seeding density (Additional file 1: Fig. S1H).

### Nutrient utilization and metabolic profile during differentiation toward the pancreatic progenitor cell fate

**Fig. 3:**
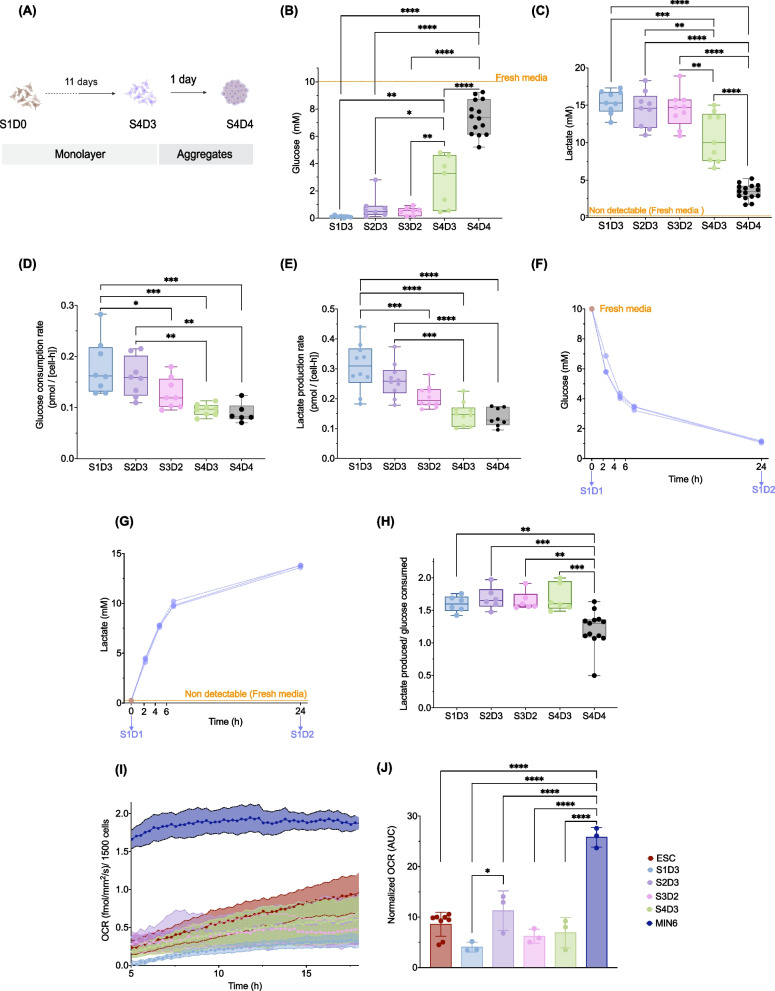
Nutrient utilization and metabolic profile during differentiation toward pancreatic progenitors. **A** Schematic of cell morphology (2D vs 3D) during four-stage differentiation. **B** Glucose concentrations (*n* = 7–14 biological replicates, one-way ANOVA with Tukey post-test; orange dash line indicates glucose concentration in fresh media) and **C** lactate concentration measured in spent media (*n* = 7–14 biological replicates, one-way ANOVA with Tukey post-test; orange dash line indicates lactate concentration in fresh media). **D** Glucose consumption rates (*n* = 5–7 biological replicates; one-way ANOVA with Tukey post-test) and **E** lactate production of S1D3 to S4D4 cells (*n* = 8–10 biological replicates; one-way ANOVA with Tukey post-test). **F** Glucose concentration and **G** lactate concentration in the spent media between S1D1 and S1D2 (*n* = 3 biological replicates); panels F-G: media were samples at 2.5, 4.5, 6.5 and 24 h after the last media change; orange dots represent concentration in fresh media. **H** Lactate yield per glucose consumed in stage-specific spent media (*n* = 6–13 biological replicates, one-way ANOVA with Tukey post-test). **I** Basal OCR and **J** Area under the curve (AUC) of stem cell derivatives, undifferentiated H1 cells and MIN6 cells (*n* = 3—4 biological replicates, one-way ANOVA with Tukey post-test). All box and whisker plots show individual points with median and interquartile range; bar charts show individual points with mean ± SD. *P < 0.05, **P < 0.01, ***P < 0.001, ****P < 0.0001. Spent media were sampled 24 ± 2 h from the previous media change unless stated otherwise; the x-axis labels on all panels denote the stage and day, e.g., S1D1 = stage 1 day 1

The growth, viability and survival of cells can be influenced by the availability of nutrients [27, 28]. To assess the stage-specific utilization of available nutrients, we measured the remaining glucose, lactate and amino acid concentrations in the spent media at the end of each stage, including the transition from monolayer to aggregates (Fig. 3A). From S1D3 to S3D2, > 90% of the glucose available was consumed with < 1 mM glucose measured in the spent media (Fig. 3B and Additional file 2: Fig. S2A). The percentage of glucose consumed between feedings decreased to ~ 60% by the end of the monolayer culture (S4D3) and ~ 25% during aggregate formation (S4D4) (Fig. 3B and Additional file 2: Fig. S2A). A significant accumulation of lactate (median = 10–15 mM) was observed during cell differentiation on a monolayer, which decreased to 3.5 mM by S4D4 (Fig. 2C). Both glucose consumption and lactate production rates steadily declined from S1D3 to S4D4 (Fig. 3D–E). Substantial glucose consumption and lactate production were observed after daily spent media sampling (Additional file 2: Fig. S2B and S2C), indicating dramatic fluctuations in the culture conditions. In fact, more frequent media sampling between S1D2 and S1D3 showed that within 6.5 h of the media change, ~ 70% of the glucose was consumed, concomitant with high lactate accumulation (Fig. 3F–G). We hypothesized that the low levels of glucose measured in the spent media may have limited cell growth, but viability remained above 80% during the presumed period of glucose deprivation (Fig. 2E). Amino acids can serve as alternative fuels to support cell growth and viability [29]. We found aspartic acid, arginine, cysteine, valine, leucine, methionine and isoleucine depleted during this time (Additional file 2: Fig. S2D). There was an accumulation of ammonia, likely due to amino acid catabolism. Glutamine and alanine were not growth-limiting as the levels in the spent media were always higher than in fresh media, likely because we supplemented with GlutaMAX™, an alanine–glutamine dipeptide. Collectively, the data are reminiscent of the Warburg effect, where cells utilize high glucose and produce high lactate in the presence of oxygen [30]; thus, we hypothesized that cells rely primarily on glycolysis between S1D3 and S4D3.

To further interrogate the metabolic phenotype of our cells during differentiation, we evaluated the lactate yield per glucose consumed and the OCR of the cells. The ratio of lactate per glucose was ≥ 1.5 until S4D3, with a significant decrease at S4D4, suggesting glycolysis as the primary energetic pathway up to S4D3 (Fig. 3H). Basal OCR of S1D3 to S4D3 cells were similar to undifferentiated H1 cells, which primarily use glycolysis [31], and ~ 2.5-fold lower than MIN6 insulinoma cells, which use OXPHOS for their energetic demands (Fig. 3I–J). Similarly, OCR during a mitochondrial stress test was lower in the stem cell derivatives as compared to MIN6 cells, irrespective of the challenge (Additional file 2: Fig. S2E). There was a steady increase in OCR/ECAR during the differentiation (Additional file 2: Fig. S2F). These results further support our hypothesis that cells rely primarily on glycolysis between S1D3 and S4D3.

### Impact of cell morphology (2D Vs 3D) on glucose utilization and lactate accumulation during the differentiation toward pancreatic progenitors

We noted significant differences in the levels of glucose consumed and lactate produced between S4D3 (monolayer) and S4D4 (aggregates) (Fig. 3B–C). As result, we investigated whether the differentiation format (2D monolayer vs. 3D aggregates) contributed to the apparent high glucose utilization and the use of glycolysis to meet energy demands. We previously demonstrated that the initial monolayer seeding density of hPSCs can affect the generation of adherent pancreatic progenitors [32]; therefore, we also tested the effect of the initial seeding concentration of aggregates (0.25e6–1e6 cells/mL) on the metabolic profile of the cells and the differentiation efficiency.

Since aggregate size can impact cell fate during differentiation [33], we formed relatively homogenous H1 aggregates (mean diameter = 130 ± 20.3 µm) using vertical wheel bioreactors (0.1 PBS-Minis) before the start of the differentiation to S4D4. Aggregate morphology was initially compact at S1D3 in most conditions (Additional file 3: Fig. S3A). However, by S2D3, aggregates were looser with a distinct blebbing or balloon phenotype, which resolved back to compacted spheres by S3D2. Surprisingly, the 0.25e6 cells/mL condition produced aggregates with a cystic appearance by S4D4, with polarized hollow cores, unlike the other conditions. The median aggregate diameter increased from ~ 130 µm to ~ 200 µm in all conditions (Additional file 3: Fig. S3B). DE fate was induced by S1D3 in all conditions based on FOXA2 and SOX17 protein levels and gene expression (Additional file 3: Fig. S3C-D). The 0.25e6 cells/mL condition had poor induction of pancreatic progenitors with ~ 30% NKX6.1+/PDX1+ cells and low gene expression of *NKX6.1* and *PDX1* (Additional file 3: Fig. S3E). Notably, there was high *CDX2* expression*,* suggesting these cells may be intestinal or enterochromaffin cell precursors. In contrast, the remaining conditions had higher expression of *NKX6.1, PDX1* and *NEUROD1* (Additional file 3: Fig. S3F), as well as a bimodal population of NKX6.1+ cells (Additional file 3: Fig. S3E). These results highlight that initial seeding concentration can affect the differentiation of hPSC aggregates toward the pancreatic progenitor fate.

As cell aggregates increase in size, they may experience morphogen gradients and diffusion limitations. Evenly seeded cells on a monolayer presumably have easier and more consistent access to nutrients in the media than cells near the center of 3D aggregates. We compared the nutrient utilization of cells differentiating in 2D and 3D formats. Viability was maintained > 80% regardless of format or initial seeding concentration, and the cell yield by S4D4 was ~ 1.5 to 2e6 cells/mL in most 3D cases compared to ~ 3e6 cells/mL in the monolayer control (Additional file 3: Fig. S3G). When cells differentiated in 3D suspension reached concentrations of 3–4e6 cells/mL, there was a subsequent decline in cell counts, perhaps indicative of a maximum cell loading capacity in our bioprocess. The lowest initial condition tested (0.25e6 cells/mL) reached a maximum cell concentration by S1D3 and had the lowest yield of S4D4 cells per input hESC despite maintaining high cell viability (> 90%). Daily spent media samples from 3D differentiation for the first four stages were collected and analyzed for glucose and lactate content. In most 3D conditions, ~ 70–90% of glucose was utilized daily until S3D2, with ≤ 3 mM glucose left over, and the concentration of lactate in the spent media was > 12 mM, similar to the monolayer control (Additional file 3: Fig. S3H-I). To determine whether differences in the feed volume and cell number between 2 and 3D formats impacted glucose consumption and lactate production, we calculated the consumption and production rates, respectively. Cells initially seeded with 0.25e6 cells/mL had dissimilar glucose consumption rates between S2D3 and S4D4 compared to other conditions (Additional file 3: Fig. S3J). Notably, the 0.25e6 cells/mL had poor pancreatic progenitor induction. In contrast, The glucose consumption rates, lactate production rates and the yield of lactate produced per glucose consumed at the end of the other 3D conditions were more similar to the monolayer (Additional file 3: Fig. S3J–L). Furthermore, S4D4 aggregates generated from the monolayer control had similar glucose consumption rates (0.0811- and 0.0827 pmol/[cell-h] for S4D4 3D and 2D, respectively) and lactate production rates (0.122- and 0.147 pmol/[cell-h] for S4D4 3D and 2D, respectively) to the S4D4 monolayer cells. The data suggest that the cell format (2D vs. 3D) does not cause high glucose utilization and lactate accumulation and that glucose consumption rates may be CQAs of efficiently differentiated hPSC-derived pancreatic progenitors. Taken together, the data show that during differentiation toward pancreatic progenitors, cells rely on glycolysis regardless of format (2D vs. 3D). Secondly, the initial seeding density of hPSC aggregates can impact the generation of pancreatic progenitors.

### Impact of glucose concentration on the growth of hPSC-derived pancreatic progenitors

**Fig. 4:**
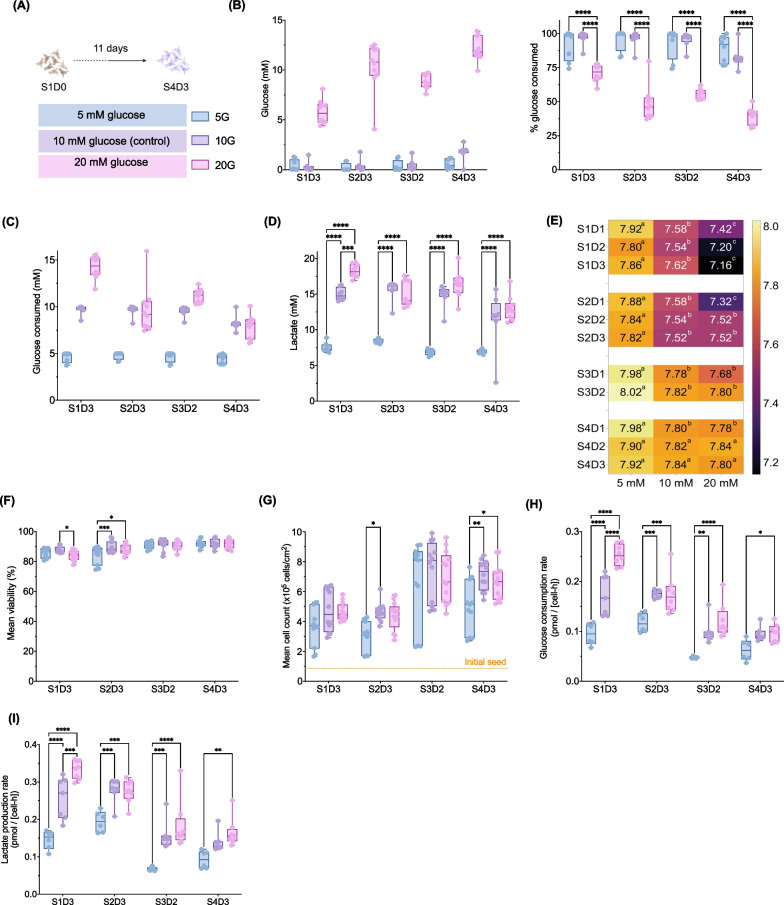
Impact of glucose concentration on the growth and the generation of hPSC-derived pancreatic progenitors. **A** Schematic of experimental design **B** Stage-specific glucose concentration and percent glucose consumed. **C** Stage-specific concentration of glucose consumed and **D** stage-specific lactate concentration in spent media and from cells cultured in media with 5 mM, 10 mM (control) or 20 mM glucose (B-D, *n* = 8–10 biological replicates; two-way ANOVA with Tukey post-test). **E** pH measured in spent media samples (*n* = 5 biological replicates and the superscripts a, b, c, are significantly different from each other by one-way ANOVA with Tukey post-test). **F** Viability and **G** cell density of the respective conditions (F-G, *n* = 11–13 biological replicates; two-way ANOVA with Tukey post-test; orange dash line = initial seeding density). **H** Quantification of stage-specific glucose consumption rates and **I** lactate production rate (H-I, *n* = 6–8 biological replicates; two-way ANOVA with Tukey post-test). All box and whisker plots show individual points with median and interquartile range; bar charts show individual points with mean ± SD. *P < 0.05, **P < 0.01, ***P < 0.001, ****P < 0.0001. Spent media were sampled 24 ± 2 h from the previous media change; S1D3 = stage 1 day 3, S2D3 = stage 2 day 3, S3D2 = stage 3 day 2, S4D3 = stage 4 day 3

Glucose is an important substrate for cell growth and proliferation [34]. The low concentrations of glucose measured in the supernatant during the first four stages suggested that the cells may be glucose deprived (Fig. 3B). Regardless of using adherent or suspension culture during differentiation to pancreatic progenitors, we observed dramatic glucose utilization. To investigate whether the glucose concentrations used in our media during the first four stages were growth-limiting, we differentiated cells with half or double the original glucose concentration (Fig. 4A). We first evaluated the cell identity and found all conditions had upregulated DE-related genes (*SOX17*, *FOXA2*) and protein levels by S1D3 (Additional file 4: Fig. S4A–B). We demonstrated that our S4D3 lifting method enriches *CDX2*^low^ cells (Fig. 2H–I). At S4D3, the 5 mM glucose (5G) condition had minimal lifting (< 10%) compared to 10- or 20 mM glucose (10G or 20G; > 70%; Additional file 4: Fig. S4C). While PP-related genes were upregulated, there were significantly fewer NKX6.1+/PDX1+ cells in the 5G-lifted monolayer by S4D3 (~ 30%) compared to 10G or 20G conditions (~ 80%) (Additional file 4: Fig. S4D). Interestingly, *PDX1* expression was comparable in all conditions, while *NKX6.*1 expression was highest in the 5G condition (Additional file 4: Fig. S4E). We then measured glucose and lactate in the spent media and found that > 80% of the glucose was consumed, with less than 0.6 mM glucose measured in most spent media samples from both 5G and 10G (Fig. 4B–C). Cells cultured in 20G consumed 40 -70% of the glucose, with ~ 6 to 12 mM glucose measured in the spent media. The 5G condition had ~ 7.5 mM lactate throughout compared to ~ 14 to 18 mM from either 10G or 20G (Fig. 4D). We observed a striking difference in the phenol red indicator as early as S1D1 (Additional file 4: Fig. S4F), likely due the variable lactate accumulation. The pH was different between all groups during stage 1, with means of 7.86, 7.62 and 7.16 for 5G, 10G and 20G, respectively (Fig. 4E). These differences gradually lessened by S4D3, suggesting that cells in 10G and 20G media had a more acidic environment earlier during differentiation compared to cells in 5G. The net lactate yield per glucose consumed was ≥ 1.3 in all conditions (Additional file 4: Fig S4G). Having determined the glucose and lactate concentrations left over in the spent media, we assessed the impact of glucose on growth kinetics and viability. There was no obvious impact of the glucose concentration on cell viability: the average viability in all conditions was > 85% (Fig. 4F). The 5G condition had ~ 1.5-fold fewer cells by the S2D3 and S4D3 compared to 10G and 20G, but there was no difference between 10 and 20G at any stage (Fig. 4G). Interestingly, we found that at S1D3, the cells modulated both their glucose consumption and lactate production rates proportional to the amount of glucose in the differentiation media (Fig. 4H–I). Thereafter, 10G and 20G rates were similar to each other and higher than the 5G rates. Taken together, the data suggest that although cells cultured in 5 mM glucose maintained high viability, this condition was growth-limiting and impacted differentiation efficiencies by the pancreatic progenitor stage. In contrast, 10 mM glucose, the standard concentration in our differentiation, was not growth-limiting. Instead, the cells consumed more glucose when the substrate was available further highlighting glucose consumption rate as a CQA and glucose concentration as a CPP of hPSC-derived pancreatic progenitors.

### Nutrient utilization of pancreatic progenitors

To determine whether glucose consumption and lactate production observed during differentiation to pancreatic progenitors were unique to the H1 cell line and our protocol, we generated H1- and H9-derived pancreatic progenitors using the STEMdiff™ pancreatic progenitor kit (referred to as kit-derived). Of note, the initial seeding density, glucose concentration in the differentiation media and the duration of stages 1, 3 and 4 are different from our protocol. Despite these differences, there was a net positive cell yield and an increase in PDX1+/NKX6.1+cells by the end of stage 4. We obtained ~ 1e6 cells/cm^2^ by S4D4, the equivalent of ~ 4 S4D4 cells per input hESC in both cell lines (Additional file 5: Fig. S5A-B). On average, we had 90% PDX1+ cells, of which 70% were PDX1+/NKX6.1+ (Additional file 5: Fig. S5C). Approximately 5 mM glucose was measured in the spent media by S1D2 in both cell lines, and between S2D1 and S4D4, there was ~ 5 to 10 mM or 8–15 mM glucose remaining for H1 and H9 lines, respectively (Additional file 5: Fig. S5D). This represented ~ 70% glucose consumption during stage 1 and between 30 and 60% glucose consumed between S2D1 and S4D4 (Additional file 5: Fig. S5E). The lactate accumulated in the spent media during the four-stage differentiation ranged between 10 and 25 mM (Additional file 5: Fig. S5F). Finally, the average lactate per glucose consumed was ~ 1.5 (Additional file 5: Fig. S5G). These results demonstrate that the substantial glucose utilization and lactate accumulation are attributes of the in vitro differentiation of hESC to pancreatic progenitors and are not specific to our differentiation protocol or cell line.

### Minimizing the formation of megaclusters caused by fusion to reduce cell loss

**Fig. 5:**
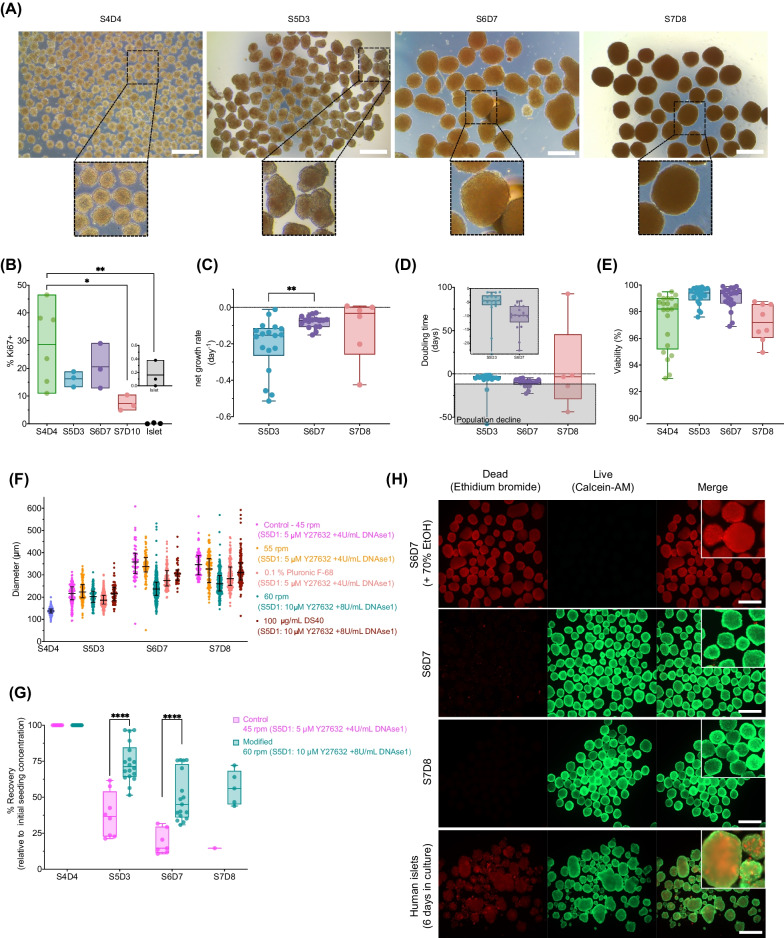
Minimizing the formation of megaclusters caused by fusion reduces cell loss between S5D3 and S6D7. **A** Representative aggregate morphology between S4D4 and S7D8, scale bar = 500 µm. **B** Ki67 positive cells (*n* = 3–6 biological replicates, 3 donor human islet preps; one-way ANOVA with Tukey post-test) based on flow cytometry. **C** Stage-specific net growth rate (*n* = 6–18 biological replicates; one-way ANOVA with Tukey post-test), **D** doubling times *n* = 5–18 biological replicates; one-way ANOVA with Tukey post-test) and **E** viability quantification using NC-200 (*n* = 8–21 biological replicates; one-way ANOVA with Tukey post-test). **F** Aggregate size distribution after testing different strategies to decrease cluster fusion (plot shows individual aggregates with median and interquartile range). **G** The modified protocol, which results in smaller aggregates, had significantly higher cell recovery during S5D3 and S6D7 (*n* = 8- 18 biological replicates; Mixed-effects analysis with Sidak post-test). **H** Representative viability of S6D7, S7D8 aggregates and donor human islets using calcein-AM and ethidium bromide, scale bar = 500 µm. (All box and whisker plots show individual points with median and interquartile range. *P < 0.05, **P < 0.01, ***P < 0.001, ****P < 0.0001. S4D4 = stage 4 day 4, S5D3 = stage 5 day 3, S6D7 = stage 6 day 7, S7D8 = stage 7 day 8, S7D10 = stage 7 day 10

We further differentiated the pancreatic progenitors into islet-like insulin-producing cell aggregates using vertical wheel bioreactors (PBS-Minis) and examined their morphology and growth kinetics. There was a substantial increase in the aggregate diameter from ~ 130 µm at S4D4 to ~ 400 µm at S7D8 (Fig. 5A). Interestingly, the peak aggregate diameter distribution was at S6D7, not S7D8. There was evidence of aggregate fusion by S5D3, resulting in the formation of megaclusters (several individual clusters tightly fused together). Despite minimal signs of proliferation (average of < 20% Ki67 + cells) and high viability (> 90%), there was cell loss from S5D3 onward as indicated by negative net growth rates (Fig. 5B–E).

Large aggregates may lead to poor-quality cells due to hypoxia and necrosis in the core [35]. We investigated the effects of several strategies for reducing cluster fusion, based on results from previous studies [36–38]. Specifically, we tested chemical (addition of surfactants DS40, Pluronic F68) and bioprocess (increased agitation rate) modifications to reduce the formation of megaclusters up to S6D7. We also increased the concentration of DNase I and Y-27632 at S5D1 only (the time of seeding aggregates into the bioreactor) in an attempt to prevent stickiness and improve cell survival following the transition from static to dynamic suspension cell culture. Increasing the agitation rate from 45 to 55 rpm did not decrease the aggregate diameter distribution (Fig. 5F). This is likely because the aggregates were pre-formed before seeding into the bioreactors or because the increase in agitation rate was not sufficient enough to have the desired effect. Adding 0.1% Pluronic F68 or 100 µg/ml DS40 (in the presence of 8 U/mL DNase I and 10 m M Y-27632 at S5D1) resulted in smaller aggregates by S7D8 (Fig. 5F). We found that by increasing agitation to 60 rpm (with 8 U/mL DNAse I and 10 µM Y-27632), the median aggregate size was reduced to ~ 260 µm compared to the control condition of 45 rpm yielding ~ 360 µm aggregates. These modifications improved cell recovery to ~ 74% at S5D3 (vs. ~ 38% control) and ~ 50% at S6D7 (vs. ~ 19% control) (Fig. 5G). By S6D7, INS, GCG and SST were similar in clusters made with the control or modified conditions (Additional file 6: Fig. S6A). Mean cell recovery at S7D8 was ~ 56%. This modified condition was used in subsequent studies. Notably, for each experimental condition tested, we found that the median aggregate diameter peaked at S6D7. We evaluated hypoxia in our aggregates using Image-iT™ Green hypoxia reagent after validating the utility of the dye (Additional file 6: Fig. S6B). Notably, we observed minimal hypoxia at S6D7 (Additional file 6: Fig. S6C) with a substantial increase by S7D8. Nevertheless, the viability of the clusters remained high (Fig. 5H). Based on S4D3 (Fig. 2D) and S7D8 cell yields (Fig. 5G), we generated ~ 3.5 S7D8 cells per input hESC.

### Nutrient utilization, hormone secretion and metabolic profile during late-stage (S5D3–S7D8+) differentiation

**Fig. 6:**
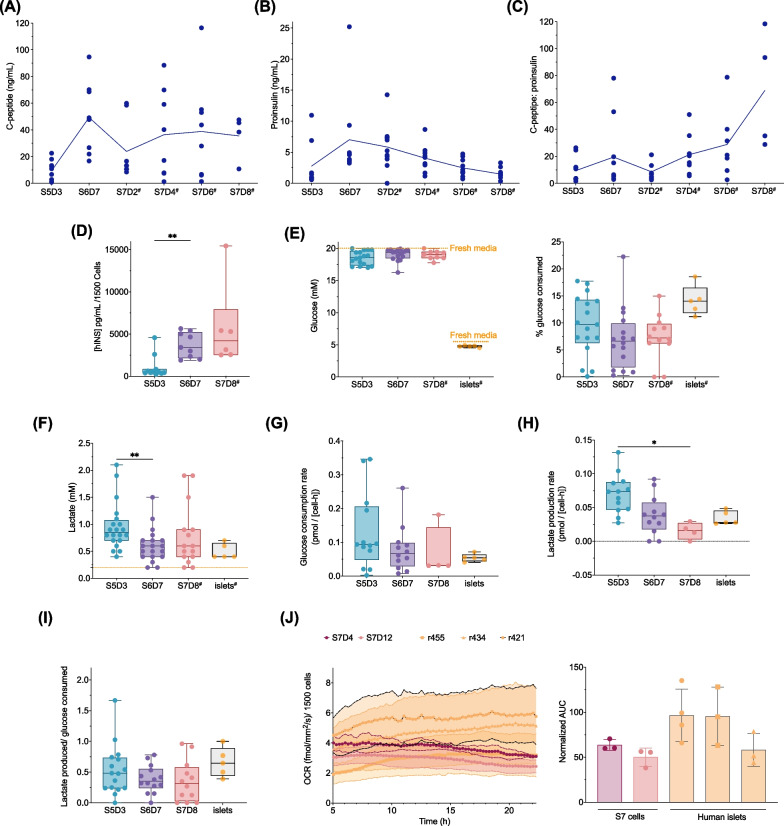
Nutrient utilization and metabolic profile during late-stage (S5D3–S7D8) differentiation toward hESC-derived insulin-producing cells indicates the cells rely primarily on oxidative phosphorylation similar to donor human islets. **A** C-peptide, **B** proinsulin and **C** C-peptide to proinsulin ratio measured in spent media samples. For panels A-C, the solid line = mean (*n* = 4–9 biological replicates). **D** Intracellular insulin content (*n* = 6–11 biological replicates; mixed-effects analysis with Tukey post-test). **E** Glucose concentration with percent glucose consumed (*n* = 9–17 biological replicates, 5 donor human islets; orange dash line indicates glucose concentration in fresh media; mixed-effects analysis with Tukey post-test). **F** Lactate concentration measured in stage-specific spent media (*n* = 15–20 biological replicates, 5 donor human islet preps; orange dash line indicates lactate concentration in fresh media; mixed-effects analysis with Tukey post-test). For panels A-F, # indicates the spent media were sampled 48 ± 2 h from the previous media change; otherwise, spent media were sampled 24 ± 2 h from the previous media change. **G** Glucose consumption rates, **H** lactate production rates (G-H *n* = 4–13 biological replicates, 5 donor human islets; mixed-effects analysis with Tukey post-test). **I** lactate yield per glucose consumed in stage-specific spent media (*n* = 12–15 biological replicates, 5 donor human islet preps; mixed-effects analysis with Tukey post-test). **J** Oxygen consumption rates (*n* = 3 biological replicates and 3 donor human islets). All box and whisker plots show individual points with median and interquartile range; bar charts show individual points with mean ± SD. *P < 0.05, **P < 0.01, ***P < 0.001, ****P < 0.0001. Spent media were sampled 24 ± 2 h from the previous media change unless indicated otherwise; S5D3 = stage 5 day 3, S6D7 = stage 6 day 7, S7D4 = stage 7 day 4, S7D8 = stage 7 day 8, S7D12 = stage 7 day 12

We assessed the nutrient utilization of our stem cell-derived endocrine cells between stages 5 and 7 by measuring metabolite levels in spent media samples. Unlike the complete daily media changes between stages 1 and 6, stage 7 and human islet cultures have media changes every 48 h. There was an accumulation of C-peptide and proinsulin in the media which peaked at S6D7 (Fig. 6A–B and Additional file 7: Fig. S7A) despite the cell losses during endocrine differentiation (Fig. 5F). Similar C-peptide accumulation was observed following *PAX4* overexpression in hPSC-derived pancreatic progenitors [39]. There was a drop in C-peptide after S6D7 in most differentiations regardless of glucose concentration during S7 or extending the duration of S6 (Fig. 6A and Additional file 7: S7B). Nevertheless, the C-peptide-to-proinsulin ratio in the spent media increased through to S7D8, and the intracellular insulin content which peaked at S6D7 was maintained at S7D8 (Fig. 6C–D). Taken together, we propose that accumulated C-peptide in the media is a QTPP, and the drop during S7 may be indicative of a switch from primarily constitutive to more regulated hormone secretion. We also measured the levels of glucose and lactate remaining in the spent media. At S5D3, an average of  >18.5 mM glucose remained in the spent media, the equivalent of consuming just 7.5% of the available glucose (Fig. 6E). At S6D7 and S7D8, we observed similar low utilization of glucose. We also found that human islets cultured in 5.5 mM glucose-containing media had ~ 4.8 mM measured in the spent media (~ 13% glucose consumed). On average, lactate levels in the spent media were < 1 mM regardless of the stage or human islet donor (Fig. 6F). There was a gradual decrease in glucose consumption and lactate production rates between S5D3 and S7D8, with values approaching that of cultured human islets (Fig. 6G–H). Based on the low utilization of glucose during the later stages of the differentiation, glucose is unlikely a limiting nutrient from stage 5 onward.

The coupling of glucose metabolism and OXPHOS is critical for human islet function [40]. In contrast to the high glucose utilization and lactate production observed during the first four stages of the differentiation (Fig. 3), these parameters were both reduced in stem cell-derived endocrine cell types. We hypothesize that during our differentiation, the cells make a metabolic shift from glycolysis to OXPHOS by S5D3. Lactate yield per glucose consumed was, on average, < 0.7 at S5D3 to S7D8 (Fig. 6I). While there was no significant difference between S7 cells and human islets, S7 OCR was generally lower (up to ~ 1.5-fold lower) (Fig. 6J); however, we noted that basal OCR of donor islets had more variability than the stem cell derivatives. These results, in combination with minimal lactate production, suggest that S7 cells rely less on glycolysis as the primary energy source as compared to S1-S4 cells.

### Characterization of cells during the endocrine stages of differentiation

**Fig. 7:**
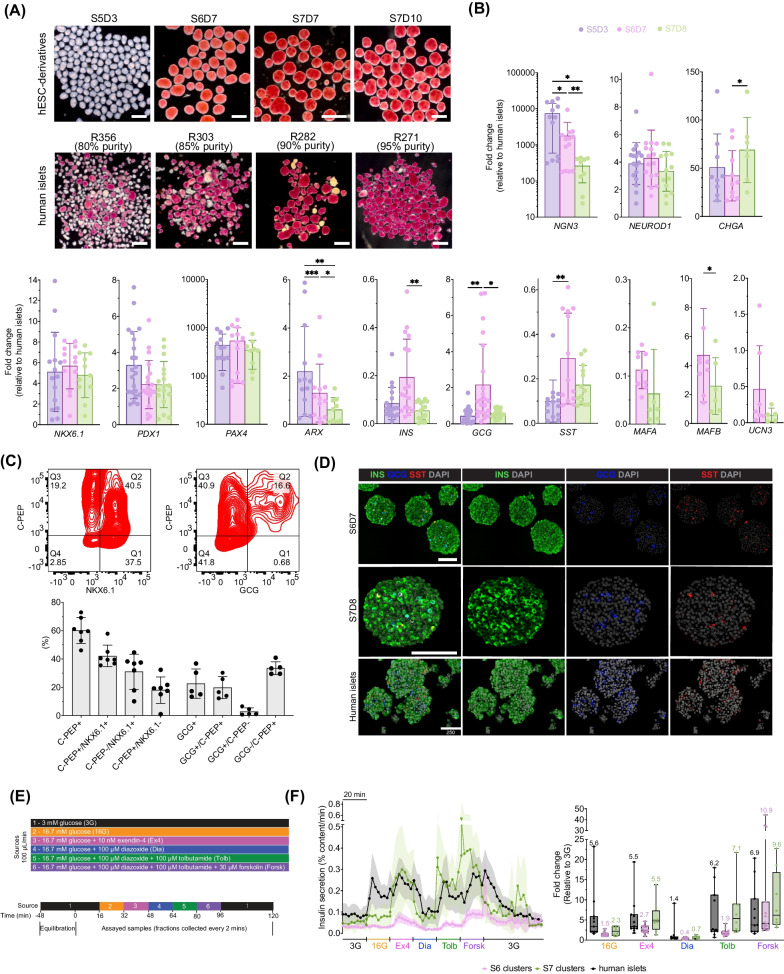
Characterization of S5D3- S7D10 (later-stage) clusters. **A** Dithizone staining of late-stage aggregates and various donor human islets, scale bar = 500 µm **B** gene expression profile of later-stage cells relative to donor human islets, scale bar = 500 µm (*n* = 13–24 biological replicates; mixed-effects analysis with Tukey post-test, *MAFA, MAFB,* *UNC3*: paired two-tailed t-test). **C** Flow cytometry of S6D7 aggregates with the indicated markers. **D** Representative INS, GCG, SST immunohistochemistry staining of S6D7, S7D8 aggregates and human islets, scale bar = 250 µm. **E** Schematic of perifusion protocol. **F** Dynamic human insulin secretion and fold change in response to various secretagogues (*n* = 15 stage 6 biological replicates, *n* = 8 biological replicates stage 7, 10 donor human islet preps; line = mean, shaded area = SEM; numbers above the whisker in fold change plots indicate the mean). All box and whisker plots show individual points with median and interquartile range; bar charts show individual points with mean ± SD. *P < 0.05, **P < 0.01, ***P < 0.001, ****P < 0.0001. S5D3 = stage 5 day 3, S6D7 = stage 6 day 7, S7D8 = stage 7 day 8

We evaluated the cell clusters for staining with dithizone (DTZ, which binds zinc within insulin granules), gene expression and protein levels of selected markers and stimulated insulin secretion. Between S6D7 and S7D10, there was an increase in the intensity DTZ staining (Fig. 7A). Peak expression of *NGN3*, the master regulator of endocrine differentiation, was at S5D3 despite the absence of a notch inhibitor in the media formulation during this stage (Fig. 7B). Of note, although *NGN3* expression dropped ~ 28-fold by S7D8, *NGN3* expression was 260-fold higher than the human islet controls, while markers of beta cells maturity (*MAFA*, *UNC3*) were relatively low, suggesting that the cells we generated are still immature. Expression of endocrine and beta cell markers *CHGA*, *NEUROD1*, *PDX1* and *NKX6.1* were ~ 2–4fold higher than in islets (Fig. 7B). Most cells at S6D7 were immunoreactive for the endocrine marker synaptophysin, but not CK19 or trypsin (ductal and exocrine markers, respectively) (Additional file 8: Fig. S8A). PAX4, a transcription factor important for beta and delta cell formation, inhibits *ARX* expression, a transcription factor important for the formation of alpha and PP-cells [41]. While there was a steady decline in *ARX* expression (0.6-fold relative to islets by S7D8), *PAX4* expression was relatively steady, albeit ~ 400-fold higher than in islets. Peak expression of hormone genes *INS*, *GCG* and *SST* was at S6D7. At S6D7, there were ~ 60% C-peptide+cells, with ~ 40% C-peptide+/NKX6.1+and ~ 20% C-peptide+/GCG+ (Fig. 7C). Cells resembling the latter have been shown to resolve into glucagon-positive alpha cells [42–44]. INS immunoreactivity was high at S6 and S7, while GCG and SST were low (Fig. 7D).

To assess the function of the cells, we performed a dynamic perifusion assay of our stage 6 and 7 cells using several secretagogues (Fig. 7E). In pancreatic beta cells, glucose-stimulated insulin secretion involves the entry and oxidation of glucose, closure of K_ATP_ channels due to the increase in ATP/ADP, and cellular depolarization that leads to an increase in intracellular calcium through voltage-gated calcium channels, which triggers the exocytosis of insulin secretory granules (reviewed in 43). Basal insulin secretion in S6D7 and S7 was about 50% lower than in human islets (Fig. 7F). Following stimulation with high glucose, on average S6 cells had a minimal increase (1.5 ± 0.48-fold), while S7 cells had a 2.3 ± 1.1-fold increase in insulin secretion (human islets 5.6 ± 6.3-fold). The incretin hormone glucagon-like peptide-1 (GLP-1) potentiates insulin secretion [46]. In the presence of the GLP-1 mimetic exendin-4, S6 and S7 cells had further increased insulin secretion (S6 = 2.7 ± 0.93-fold; S7 = 5.5 ± 3.9-fold). Diazoxide, a compound that opens K_ATP_ channels, resulted in decreased insulin secretion in S6 and S7 cells, while closure of K_ATP_ channels with the sulfonylurea tolbutamide increased insulin secretion, as did forskolin, which increases intracellular cAMP. Finally, insulin secretion decreased to near baseline when the S6 or S7 cells were returned to basal glucose concentrations; however, some S7 cells displayed unexpected pulsatile insulin secretion for ~ 10 min during the 40-min perfusion period. Altogether, we found that S6 and S7 cell clusters mostly had both first- and second-phase insulin secretion profiles similar to human islets. Still, the percentage of insulin released was lower in S6 cell clusters, while S7 clusters were more similar to human islets. Overall, the S6 and S7 cells possess the necessary machinery for insulin secretion through the triggering pathway, but these cells are still immature compared to human islets.

## Discussion

**Fig. 8:**
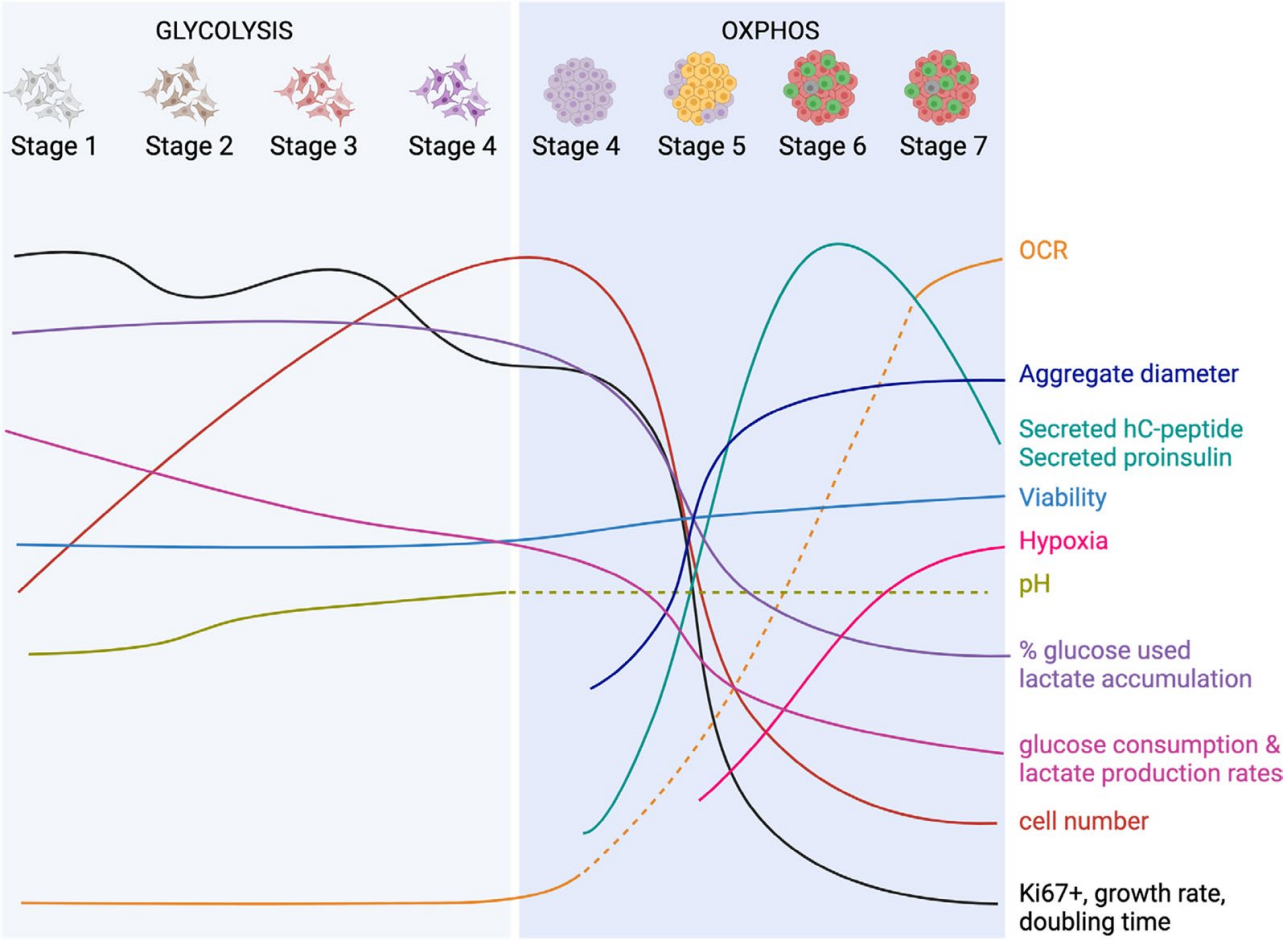
Summary of key points in this study. Graphic summary of QTPP associated with the differentiation protocol (solid line = QTPP based on data; dash line = presumed QTPP, no data collected at the indicated timepoint)

As the goal of getting cellular replacement therapies for T1D into the clinic becomes a reality, there is a need to develop robust manufacturing processes [47]. In this study, we focused on identifying and characterizing several bioprocess parameters and cell attributes during the generation of hPSC-derived insulin-producing cells (Fig. 8). We report distinct metabolic phenotypes, growth kinetics, cell function and the upregulation of stage-specific markers associated with stem cell-derived pancreatic progenitors and endocrine cells.

Generating clinically relevant numbers of cells is a desirable feature of a bioprocess with the goal of making insulin-producing cells. It is estimated that ~ 10^9^ beta cells per recipient may be needed for treating T1D [48, 49]. Defining CQAs can identify stages that may be suitable targets for improving cell yield without compromising the quality of the cells. We show that for every undifferentiated PSC, we could make ~ 7 stage 4 cells. We improved cell yield by the end of S7D8 by reducing aggregate fusion using a combination of chemical supplementation and increased agitation, decreasing aggregate size distribution. As a result, for every H1 PSC, we generated ~ 3.5 stage 7 cells. During the first four stages of differentiation, there was a steady linear increase in cell number, positive net growth rate, high viability and a decline in proliferative cells marked by Ki67+. Despite the relatively high number of Ki67+ cells, the proliferative capacity was slow: the duration of most stages was not long enough to permit the doubling of the cells. Extending the duration of each stage could generate more cells but would increase manufacturing time and costs. Aside from increasing the differentiation scale, an alternative approach to increase cell yields is to temporarily activate the proliferative capacity using small molecules and growth factors to facilitate cell expansion [50–52]. For example, Goncalves et al. reported ~ 15-fold expansion of hPSC-derived pancreatic progenitors after 10 days of culture by targeting EGF and FGF pathways [52]. These pancreatic progenitors could be passaged 20 times, without detectable karyotypic abnormalities (based on G-banding), and could be cryopreserved or further differentiated to endocrine cells.

Significant cell losses were observed during the endocrine stages of our differentiation protocol (Fig. 5F). Factors that could contribute to cell loss may include: (1) inadequate process parameters, such as the agitation rate, that are harmful to the cells, (2) cellular secretion of deleterious factors that trigger cell death, (3) an inherent feature of cells at this developmental stage resulting in death, (4) negative selection against a subset of unfit or non-competitive cells, or (5) other unidentified factor(s). By using both chemical and mechanical strategies to reduce aggregate size distribution, we improved cell yield by 1.9-fold by S5D3 and 2.6-fold by S6D7. Our approach to reducing fusion and the formation of megaclusters suggests that a combination of suboptimal agitation and the presence of megaclusters partially contributed to the cell loss. In particular, larger clusters are susceptible to a necrotic core, hypoxia and diffusion limitation when consuming soluble nutrients or excreting waste products. Part of the cell loss may be attributed to the endocrine fate of the cells. Dror et al*.* noted that overexpression of the endocrine master regulator *NGN3* induced apoptosis in adult human and mouse islets [53]. Furthermore, *Ngn3* inhibits proliferation in mouse endocrine progenitors by increasing cdkn1a [54, 55]. Peak *NGN3* expression was observed at S5D3 with our protocol, and although it significantly decreased over time, it was still high relative to human islets and may be a target to improve the cell yield.

We show efficient DE and pancreatic progenitor differentiation based on SOX17+/FOXA2+ cells and NKX6.1+/PDX1+ cells, respectively. We obtained a similar percentage of NKX6.1+/PDX1+ cells (~ 70%) by the end of stage 4 when differentiating H1 and H9 cells with the STEMdiff™ pancreatic progenitor kit. While these markers are routinely used in the field, these markers alone, while informative [56–59], are not sufficient to predict the formation of pancreatic progenitors or glucose-responsive insulin-producing cells. Screening studies have identified markers such as CD177 [60], GP2 [61–63], CD49A [23] and ENTPD3 [64] within seemingly homogenous populations that may better predict differentiation outcomes in conjunction with routine markers. These markers have been targeted and enriched with variable percent recoveries that may not be practical for large-scale manufacturing of hPSC-derived endocrine cells.

Aggregates made with our protocol contain the 3 most abundant endocrine cells found in human islets: insulin-producing beta cells, glucagon-producing alpha cells and somatostatin-producing delta cells. GSIS, although minimal at S6, increased by S7. Our data suggest that the machinery required for the triggering and amplifying pathway mediating GSIS [65] is present in our stem cell derivatives. However, the magnitude of GSIS in our S7 cells is still lower than in human islets. Prior studies have implicated improper glucose metabolism [66] as one bottleneck to acquiring comparable, if not better, GSIS in hPSC derivatives than human islets. Furthermore, extended cell culture [64], reorganization of cytoarchitecture [8], cluster reaggregation in concert with TGF-β modulation [9], or with beta cell enrichment [10] can improve the functional maturity of hPSC-derived beta cells. The final cells generated with our protocol have an immature phenotype based on the low expression of maturation markers, *MAFA, UCN3* and high expression of *MAFB.* This immature phenotype of the final stages is not unique to this protocol [6, 8–10]. Other studies showed continued upregulation of maturation makers and downregulation of disallowed genes and proliferative cells over time post-implant in diabetic or healthy rodents [5, 6, 67]. Since mature beta cell function can be achieved post-implant, generating pancreatic progenitors or immature beta cells may be adequate, particularly if maturation comes at the cost of extended culture and the associated cell losses. However, fully functional cells are still preferable for in vitro studies, disease modeling and drug screening.

Naïve ESCs primarily rely on glycolysis, whereas somatic cells typically rely on OXPHOS for their energetic needs [68, 69]. In an attempt to identify growth-limiting nutrients during the differentiation, we discovered that the cells rely primarily on glycolysis earlier in the differentiation (up to the pancreatic progenitor stage) and then switch to OXPHOS. Similar metabolic shifting has been reported during cardiomyocyte differentiation [70], resting vs stimulated Tregs [71, 72] and primed vs naïve ESCs [73]. Cells between stages 1 and 4 had high glucose consumption, high lactate production and accumulation, hallmarks of the Warburg effect and low OCR when cultured at 21% O_2_. We also determined this phenotype to be cell fate-dependent and not based on the cell culture platform used for the differentiation (2D vs 3D). The cells maintained high viability despite low levels of glucose remaining in the spent media, which was apparently not growth-limiting. During these stages of differentiation, the cell numbers increase, similar to other reports [11, 12]. While OXPHOS generates higher amounts of ATP than glycolysis, high glycolytic rates can produce ample energy for rapidly proliferating cells. Furthermore, these proliferating cells may be sensitive to OXPHOS-generated ROS, which can be detrimental to numerous biosynthetic processes and cell survival. Although ESCs rely on glycolysis, they have functional yet immature mitochondria [74]. Lv et al. found that mitochondria homeostasis is important as cells exit pluripotency into DE over 5 days [75]. DE cells had more mature mitochondria and lower lactate content than PSCs. However, the OCR of PSCs and DE cells were similar, consistent with our findings, and so were ROS levels.

Data suggest that some stem cell-derived endocrine cells can exhibit mature mitochondria similar to those found in human islets [10]. In beta cells, pyruvate generated by glucose metabolism is shuttled into the mitochondria for further oxidation [76]. Consistent with our findings, hPSC-derived pancreatic progenitors have lower OCR compared to human islets [77], while hPSC-derived insulin-producing cells have more similar OCR to human islets [10]. It is important to note that glucose concentrations in our basal stage 7 media and human islet media are different (20 mM vs. 5.5 mM, respectively), and 20 mM glucose would typically further increase OCR in human islets [10]. Nevertheless, during the endocrine stages of differentiation, the cells used < 20% of the glucose in the media and accumulated < 1.5 mM lactate, if any, similar to human islets. Lactate yield per glucose was similar to human islets and lower than in stages 1–4, indicative of a metabolic shift from glycolysis to OXPHOS. Even still, Davis et al. showed a bottleneck in glucose metabolism in hPSC-derived insulin-producing cells generated using their protocol due to the lack of PECK-M activity, which could impact GSIS [66].

To the best of our knowledge, this is the first report to demonstrate glucose utilization and lactate accumulation in hPSC-derived derivatives during differentiation into insulin-producing cells, and we propose them as critical quality attributes (CQAs) that should be included in quality target product profiles (QTPP). Cells utilized 40–99% of the glucose in the media during the first four stages toward pancreatic progenitors. Between S1D1 and S4D3, H1-derived cells utilized the majority of the available glucose, ranging from ~ 70% to 99%, regardless of the protocol employed (either our protocol or the STEMdiff™ pancreatic progenitor kit). H9-derived S1D1 to S4D3 cells generated with the STEMdiff™ pancreatic progenitor kit used ~ 40% to 60% of the glucose during the same period. However, during the endocrine stages, the overall glucose utilization by H1-derived cells dropped to < 8% of the glucose present in the media. The feeding strategy of stem cell-derived endocrine cell types may be modified to a fed-batch method where relevant growth factors and small molecules are spiked into the bioreactor daily without a complete media change for a least 48 h. This feeding strategy would lower the burden of the operator to perform daily changes, minimize the risk of contamination due to reduced handling and lower the overall manufacturing costs. Furthermore, tracking parameters such as glucose and insulin levels in the media that do not require sacrificing cells could be amenable to in-line monitoring with process analytic technologies such as Raman spectroscopy [16].

We found that glucose consumption rates and lactate production rates are CQAs that are influenced by both glucose concentrations and cell fate. Cells that failed to differentiate into pancreatic progenitors efficiently had drastically different profiles (Additional file 3: Fig S3J–K). Furthermore, adherent cells differentiated with 5 mM glucose-containing media (Fig. 3) and PSC aggregates differentiated in 10 mM glucose-containing media with an initial cell concentration of 0.25e6 cells/mL (Additional file 3: Fig. S3) both had mostly lower lactate levels accumulated in the spent media (~ 8 mM vs. ~ 15 mM in other conditions). Both conditions failed to efficiently differentiate to pancreatic progenitors. We suspect that the relatively acidic environment resulting from lactate accumulation may be beneficial, if not necessary, for successful PP differentiation. However, further experiments are required to interrogate this hypothesis. Reporting these process parameters with current and future differentiation protocols could provide important information about their utility in the manufacturing process.

There are several limitations to this study. First, two embryonic cell lines (H1 and H9) and two protocols were used for the differentiations. While we believe the general trends observed could apply to other cell lines, it is widely known that most differentiation protocols have variable efficiencies when applied to various lines. Factors such as passage number, propagation methods, reagent sources, concentrations of nutrients (e.g., glucose) and personnel expertise can also impact the differentiation. In addition, only basal OCR of stage 7 or human islets, each of which was cultured in 20 mM or 5.5 mM glucose-containing media, respectively, was considered in this study. Further experiments are needed to evaluate both cell types under similar glucose conditions as others have done [10]. Furthermore, it is unclear the consequence of disrupting and reseeding adherent cells (stages 1–4) before measuring basal OCR. However, the stem cell derivatives, MIN6 cells and undifferentiated controls were handled in a similar manner. Finally, there is still the potential need to bridge the gap between using GMP vs non-GMP compliant reagents and cell lines with the goal of developing a manufacturing process that is suitable for clinical trials.

## Conclusion

In conclusion, we report a differentiation protocol to generate hPSC-derived insulin-producing cells where we summarize QTPPs and characterize numerous CQAs and CPPs. We also illustrate the utility of PBS-Mini bioreactors to differentiate hPSCs into pancreatic cell types. There is an increase in cell number up to stage 4 while cells are lost between stages 5 and 7. Cells primarily rely on glycolysis up to stage 4 then switch to OXPHOS. There is a gradual decrease in both glucose consumption and lactate production rates over the course of the differentiation. Further, glucose concentration and agitation rates were identified as process parameters that impact glucose consumption rate and cell recovery, respectively. Our findings contribute knowledge to the field of cell replacement therapy and the manufacture of hPSC-derived islet-like clusters.

### Supplementary Information

**Additional file 1**. **Figure S1**: Morphology, doubling times and representative flow cytometry plots during the first four stages of differentiation. (A) Representative morphology of the cell monolayer from S1D3 to S4D3, scale bar= 500 µm. (B) Quantification of stage-specific doubling time, where a negative value indicates a decline in the cell population (n= 13-15 biological replicates; box and whisker plots show individual points with median and interquartile range, one-way ANOVA with Tukey post-test). (C) Viability of undifferentiated ESCs (n= 22 independent replicates). (D) Representative flow cytometry plots of ES, S1D3 cells showing gating strategy using the appropriate isotype controls (blue). (E) Viability of lifted S4D3 cells (n= 22 independent replicates). (F) Representative flow cytometry plots of S4D3 and S4D4 cells showing gating strategy using the appropriate isotype controls (blue). (G) Aggregation efficiency of AggreWell^TM^ vs. PBS-Mini. The lines indicate paired experiments using the same input S4D3 cells to seed both AggreWell^TM^ and PBS-Mini (paired two-tailed t-test; n= 8 biological replicates note that one AggreWell^TM^ point is repeated from Fig. 1K for comparison). (H) Morphology and diameter of S4D4 aggregates formed with PBS-Minis using the indicated seeding density, scale bar= 500 µm (n= 399-479 aggregates/group; plot shows individual aggregates with median and interquartile range). S1D3 = stage 1 day 3, S2D3 = stage 2 day 3, S3D2 = stage 3 day 2, S4D3 = stage 4 day 3, S4D4 = stage 4 day 4

**Additional file 2**. **Figure S2**: Metabolites and OCR during the first four stages of differentiation. (A) Percent glucose consumed at the end of each stage (n= 7-14 biological replicates). (B) Glucose concentration and (C) lactate concentration measured in spent media sampled daily from S1D1 to S4D4 (For panels B and C, solid line= change in glucose or lactate concentration ~24 h after media change, dash line= replacement of spent media with fresh media; n= 1 biological replicate). (D) Percentage of amino acids in spent media relative to fresh media during the first four stages of differentiation (n=3 biological replicates; glutamine concentration in spent media shown as it was undetectable in fresh media). For panels A-D, spent media were sampled 24 ± 2 h from the previous media change; (E) Mito stress test OCR and (F) OCR/EACR of stages 1-4, undifferentiated H1 and MIN6 cells (n= 3-4 biological replicates; one-way ANOVA with Tukey post-test. a, b, c, d and e are significantly different from one another). FCCP=carbonyl cyanide-4-(trifluoromethoxy) phenyl hydrazone; Rot/AA= rotenone/antimycin A. S1D3 = stage 1 day 3, S2D3 = stage 2 day 3, S3D2 = stage 3 day 2, S4D3 = stage 4 day 3, S4D4 = stage 4 day 4

**Additional file 3**. **Figure S3**: Impact of cell culture format (2D Vs 3D) on nutrient utilization during the differentiation toward pancreatic progenitors. (A) Stage-specific morphology of all initial cell seeding concentrations tested, scale bar= 500 µm. (B) Aggregate diameter during the respective stages. (C) Flow cytometry and (D) gene expression at S1D3. (E) Flow cytometry and (F) gene expression at S4D3. (G) Cell concentration and viability of all conditions throughout the four-stage differentiation (plot shows technical replicates with mean, line= mean). (H) Daily glucose concentration in spent media and percentage of glucose consumed. (I) Daily lactate concentration in spent media (note that the monolayer control data in panels H and I are the same from Fig S2B-C and are replotted here for reference only). (J) Glucose consumption rate, (K) lactate production rates and (L) lactate per glucose consumed throughout four-stage differentiation. For panels G-L, the x-axis labels denote the stage and day, e.g., S1D1= stage 1 day 1

**Additional file 4**. **Figure S4**: Effect of glucose concentration on stage-specific markers. (A) Flow cytometry quantification of SOX17+/FOXA2+ at S1D3 with representative flow cytometry plots (n= 6 biological replicates; one-way ANOVA with Tukey post-test). (B) Gene expression profile of SOX17 and FOXA2 at S1D3 (n= 6-8 biological replicates; Friedman test with Dunn’s post-test). (C) Representative crystal violet staining before and after S4D3 lifting. (D) Flow cytometry quantification of NKX6.1+/PDX1+ at S4D3 with representative flow cytometry plots (n= 4 biological replicates; one-way ANOVA with Tukey post-test). (E) Gene expression profile of PDX1 and NKX6.1 at S4D3 (n= 5 biological replicates; Friedman test with Dunn’s post-test). (F) Representative change in phenol red pH indicator during S1D1. (G) Lactate-to-glucose ratio from cells (n = 6-8 biological replicates; two-way ANOVA with Tukey post-test). All box and whisker plots show individual points with median and interquartile range. *P< 0.05, **P< 0.01, ***P< 0.001, ****P< 0.0001. S1D3 = stage 1 day 3, S2D3 = stage 2 day 3, S3D2 = stage 3 day 2, S4D3 = stage 4 day 3

**Additional file 5**. **Figure S5**: Nutrient utilization of kit-derived pancreatic progenitors. (A) Cell density of H1 and H9 derived S4D4 cells (orange dash line= initial seeding density). (B) S4D4 cell yield per input H1 and H9 cell. (C) Flow cytometry quantification of NKX6.1+ and PDX1+ positive cells at S4D4 (n=7 for H1 derivatives-12 biological replicates). (D) Glucose remaining in spent media, (E) percentage glucose consumed and (F) lactate concentration measured in spent media between S1D1 and S4D4. (G) Lactate produced per glucose consumed between S1D1 and S4D4 (D-H: n= 7 biological replicates for H1 and n= 3 biological replicates for H9). For panels D-H, the x-axis labels denote the stage and day, e.g. S1D1= stage 1 day 1. Spent media were sampled 24 ± 2 h from the previous media change

**Additional file 6**. **Figure S6**: Staining of later-stage clusters. (A) Representative INS, GCG, SST immunohistochemistry staining of S6D7 clusters generated with the original or modified protocol (B) Hypoxia dye validation using undifferentiated H1 aggregates, scale bar= 750 µm. (C) Hypoxia staining of S6D7 clusters and human islets, scale bar= 750 µm. S5D3 = stage 5 day 3, S6D7 = stage 6 day 7, S7D4 = stage 7 day 4, S7D8 = stage 7 day 8

**Additional file 7**. **Figure S7**: C-peptide concentration in spent media. (A) C-peptide in spent media with 20 mM glucose (20G) differentiation media during stage 7 (S7). (B) C-peptide in spent media during extended periods of S6 or 5 mM or 10 mM glucose (5G or 10G) during S7. # indicates the spent media were sampled 48 ± 2 h from the previous media change; otherwise, spent media were sampled 24 ± 2 h from the previous media change. The x-axis labels denote the stage and day, e.g., S4D4= stage 4 day 4

**Additional file 8**. **Figure S8**: Immunostaining of clusters. (A) Representative cytokeratin19 (CK19), synaptophysin (SYNP) and trypsin immunohistochemistry staining of S6D7 and human islets

## Data Availability

The datasets used and/or analyzed are available from the corresponding author on reasonable request.

## Abbreviations

CPP: Critical process parameter
CQA: Critical quality attribute
hESCs: Human embryonic stem cells
hiPSCs: Human induced pluripotent stem cells
OXPHOS: Oxidative phosphorylation
QTPP: Quality target product profile
SD: Standard deviation
T1D: Type 1 diabetes
5G: 5 mM glucose-based media
10G: 10 mM glucose-based media
20G: 20 mM glucose-based media
