# Supplementary figures and images for: Metabolic switching, growth kinetics and cell yields in the scalable manufacture of stem cell-derived insulin-producing cells
Source: Stem Cell Res Ther. 2024 Jan 2;15:1. doi: 10.1186/s13287-023-03574-3 (PMC10762849; doi:10.1186/s13287-023-03574-3)

# Supplemental Figure 1

(A)

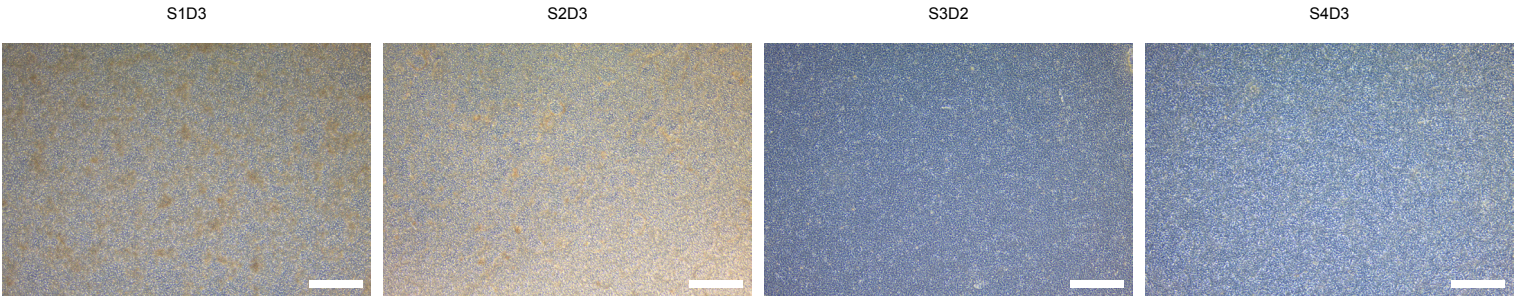

(B)

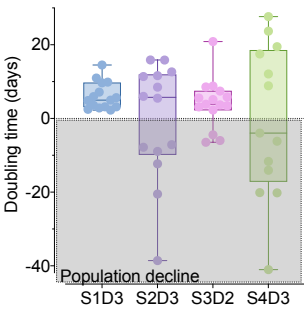

(C)

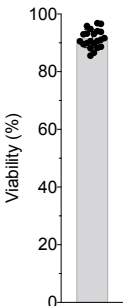

(D)

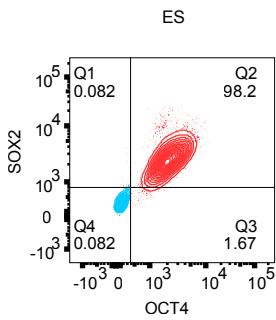

(E)

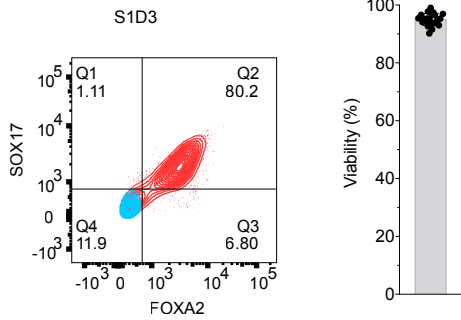

(F)

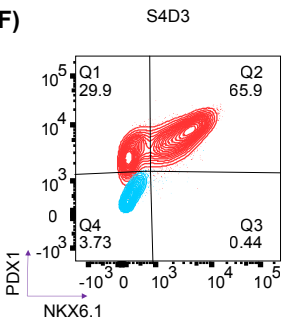

S4D4

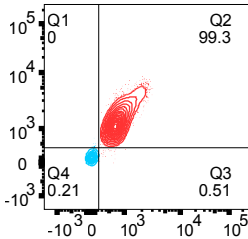

(G)

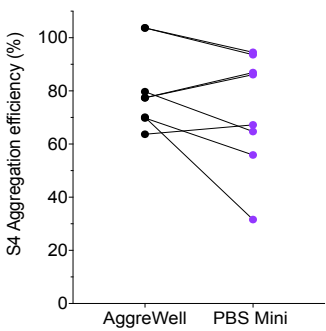

(H)

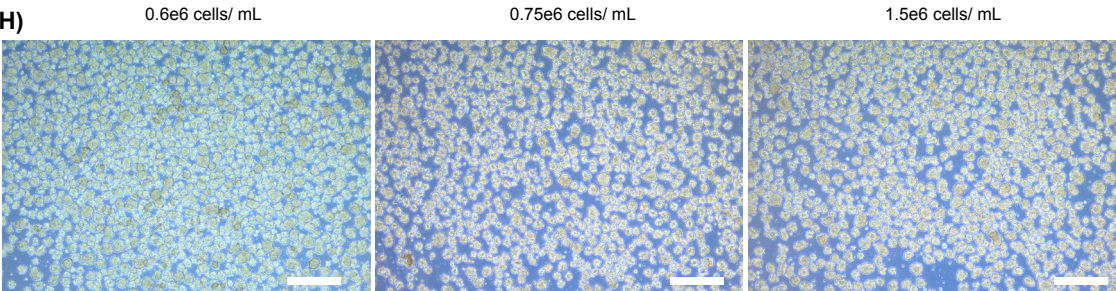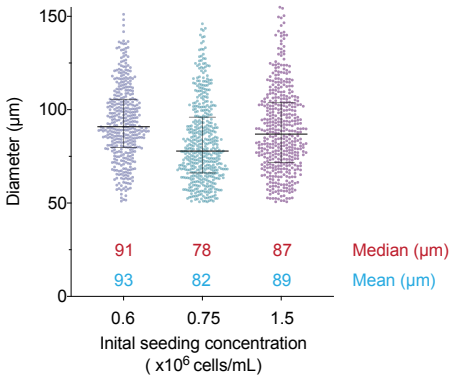

Supplement: Supplementary file 1 — Additional file 1. Figure S1: Morphology, doubling times and representative flow cytometry plots during the first four stages of differentiation. (A) Representative morphology of the cell monolayer from S1D3 to S4D3, scale bar= 500 µm. (B) Quantification of stage-specific doubling time, where a negative value indicates a decline in the cell population (n= 13-15 biological replicates; box and whisker plots show individual points with median and interquartile range, one-way ANOVA with Tukey post-test). (C) Viability of undifferentiated ESCs (n= 22 independent replicates). (D) Representative flow cytometry plots of ES, S1D3 cells showing gating strategy using the appropriate isotype controls (blue). (E) Viability of lifted S4D3 cells (n= 22 independent replicates). (F) Representative flow cytometry plots of S4D3 and S4D4 cells showing gating strategy using the appropriate isotype controls (blue). (G) Aggregation efficiency of AggreWellTM vs. PBS-Mini. The lines indicate paired experiments using the same input S4D3 cells to seed both AggreWellTM and PBS-Mini (paired two-tailed t-test; n= 8 biological replicates note that one AggreWellTM point is repeated from Fig. 1K for comparison). (H) Morphology and diameter of S4D4 aggregates formed with PBS-Minis using the indicated seeding density, scale bar= 500 µm (n= 399-479 aggregates/group; plot shows individual aggregates with median and interquartile range). S1D3 = stage 1 day 3, S2D3 = stage 2 day 3, S3D2 = stage 3 day 2, S4D3 = stage 4 day 3, S4D4 = stage 4 day 4 [file 13287_2023_3574_MOESM1_ESM.pdf]

# Supplemental Figure 2

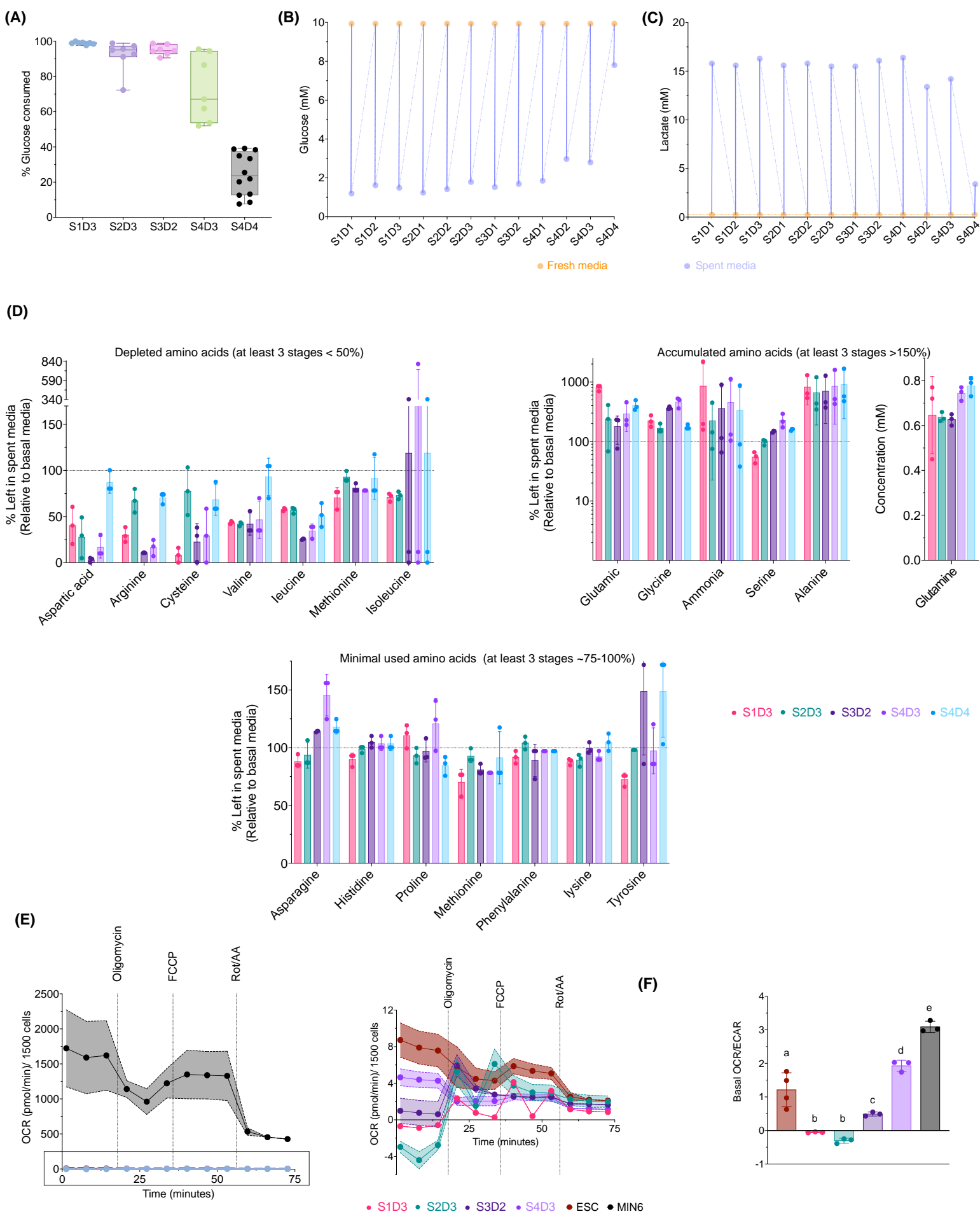

Supplement: Supplementary file 2 — Additional file 2. Figure S2: Metabolites and OCR during the first four stages of differentiation. (A) Percent glucose consumed at the end of each stage (n= 7-14 biological replicates). (B) Glucose concentration and (C) lactate concentration measured in spent media sampled daily from S1D1 to S4D4 (For panels B and C, solid line= change in glucose or lactate concentration ~24 h after media change, dash line= replacement of spent media with fresh media; n= 1 biological replicate). (D) Percentage of amino acids in spent media relative to fresh media during the first four stages of differentiation (n=3 biological replicates; glutamine concentration in spent media shown as it was undetectable in fresh media). For panels A-D, spent media were sampled 24 ± 2 h from the previous media change; (E) Mito stress test OCR and (F) OCR/EACR of stages 1-4, undifferentiated H1 and MIN6 cells (n= 3-4 biological replicates; one-way ANOVA with Tukey post-test. a, b, c, d and e are significantly different from one another). FCCP=carbonyl cyanide-4-(trifluoromethoxy) phenyl hydrazone; Rot/AA= rotenone/antimycin A. S1D3 = stage 1 day 3, S2D3 = stage 2 day 3, S3D2 = stage 3 day 2, S4D3 = stage 4 day 3, S4D4 = stage 4 day 4 [file 13287_2023_3574_MOESM2_ESM.pdf]

# Supplemental Figure 3 (1/2)

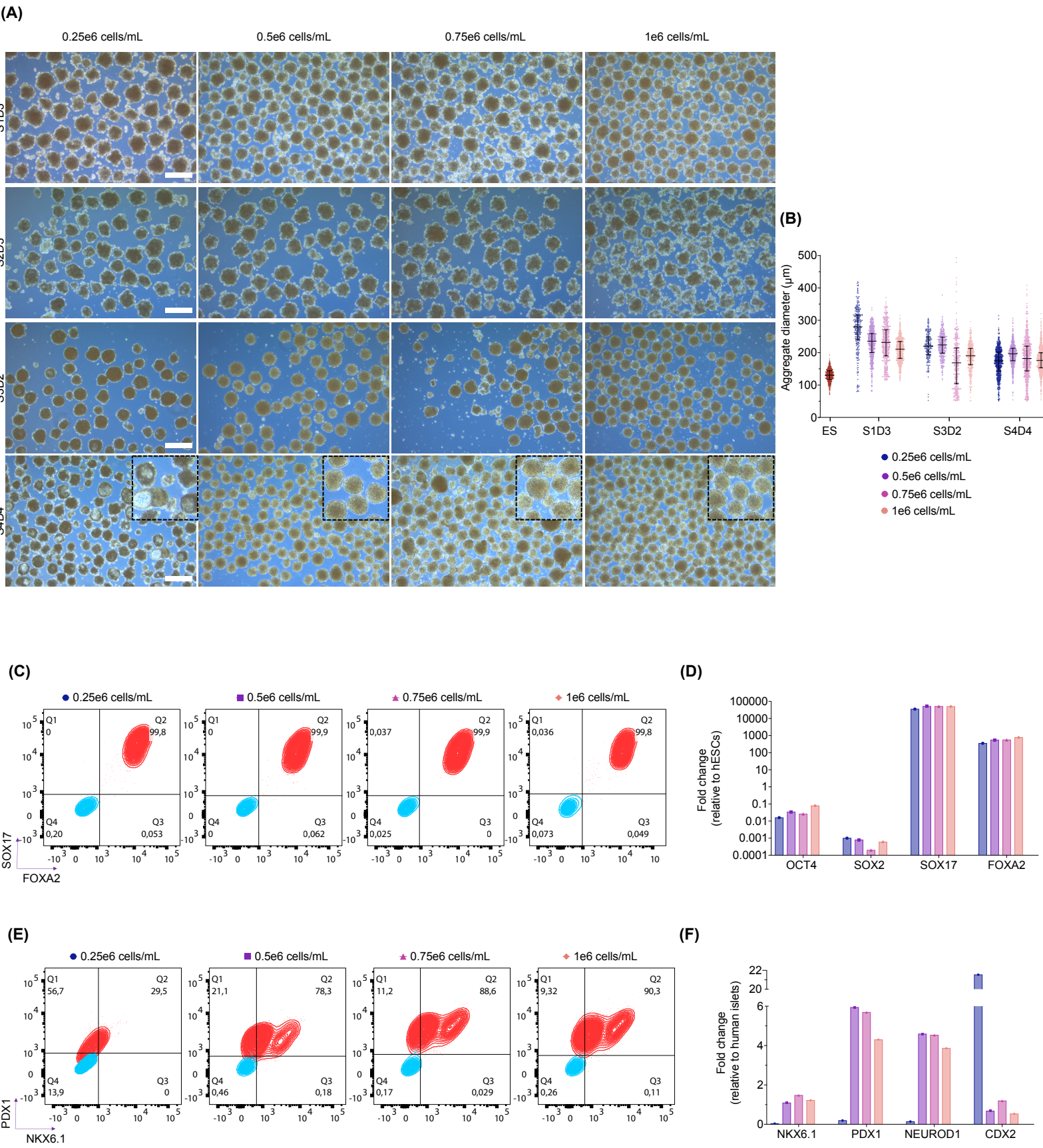

# Supplemental Figure 3 (2/2)

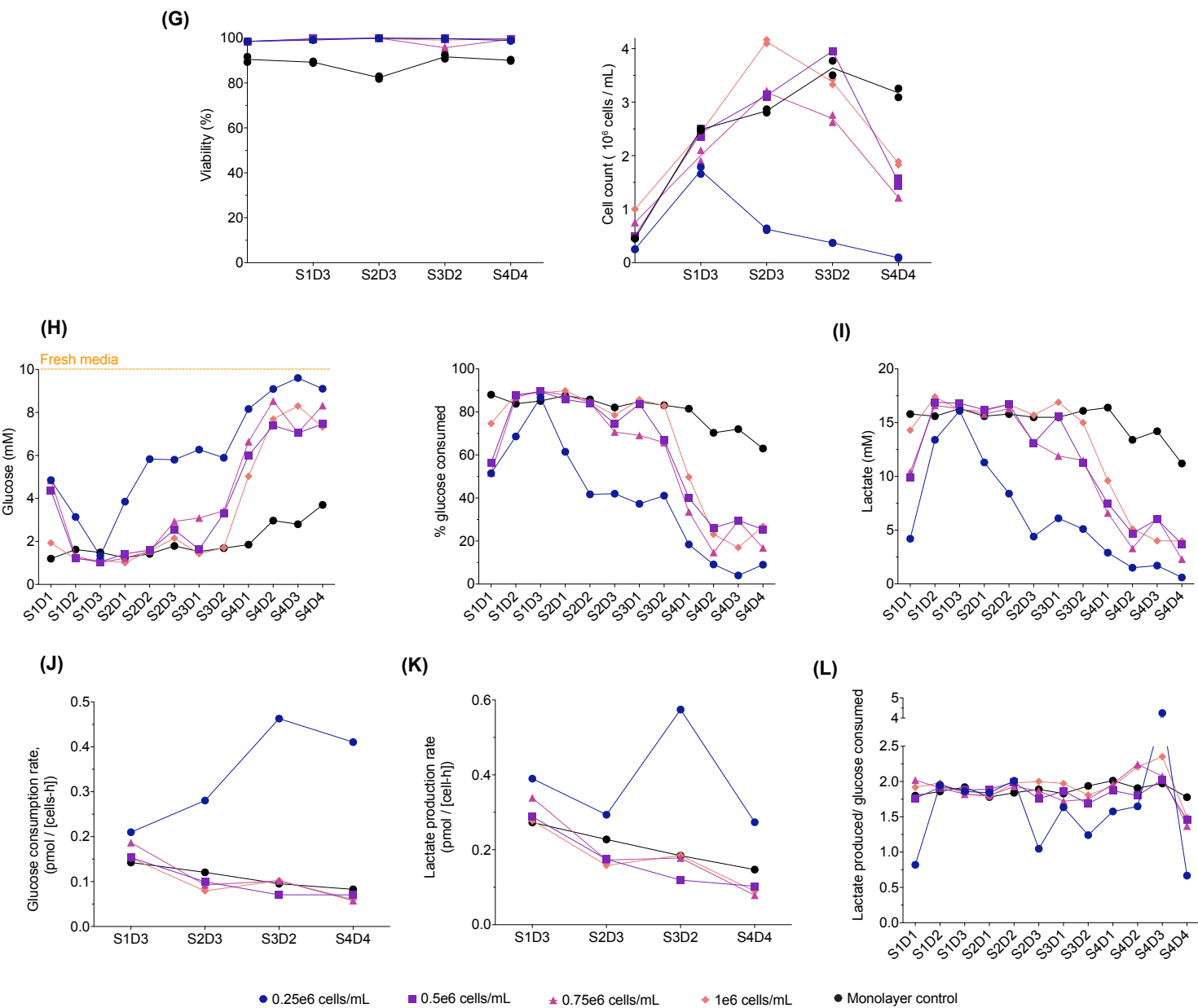

Supplement: Supplementary file 3 — Additional file 3. Figure S3: Impact of cell culture format (2D Vs 3D) on nutrient utilization during the differentiation toward pancreatic progenitors. (A) Stage-specific morphology of all initial cell seeding concentrations tested, scale bar= 500 µm. (B) Aggregate diameter during the respective stages. (C) Flow cytometry and (D) gene expression at S1D3. (E) Flow cytometry and (F) gene expression at S4D3. (G) Cell concentration and viability of all conditions throughout the four-stage differentiation (plot shows technical replicates with mean, line= mean). (H) Daily glucose concentration in spent media and percentage of glucose consumed. (I) Daily lactate concentration in spent media (note that the monolayer control data in panels H and I are the same from Fig S2B-C and are replotted here for reference only). (J) Glucose consumption rate, (K) lactate production rates and (L) lactate per glucose consumed throughout four-stage differentiation. For panels G-L, the x-axis labels denote the stage and day, e.g., S1D1= stage 1 day 1 [file 13287_2023_3574_MOESM3_ESM.pdf]

# Supplemental Figure 4

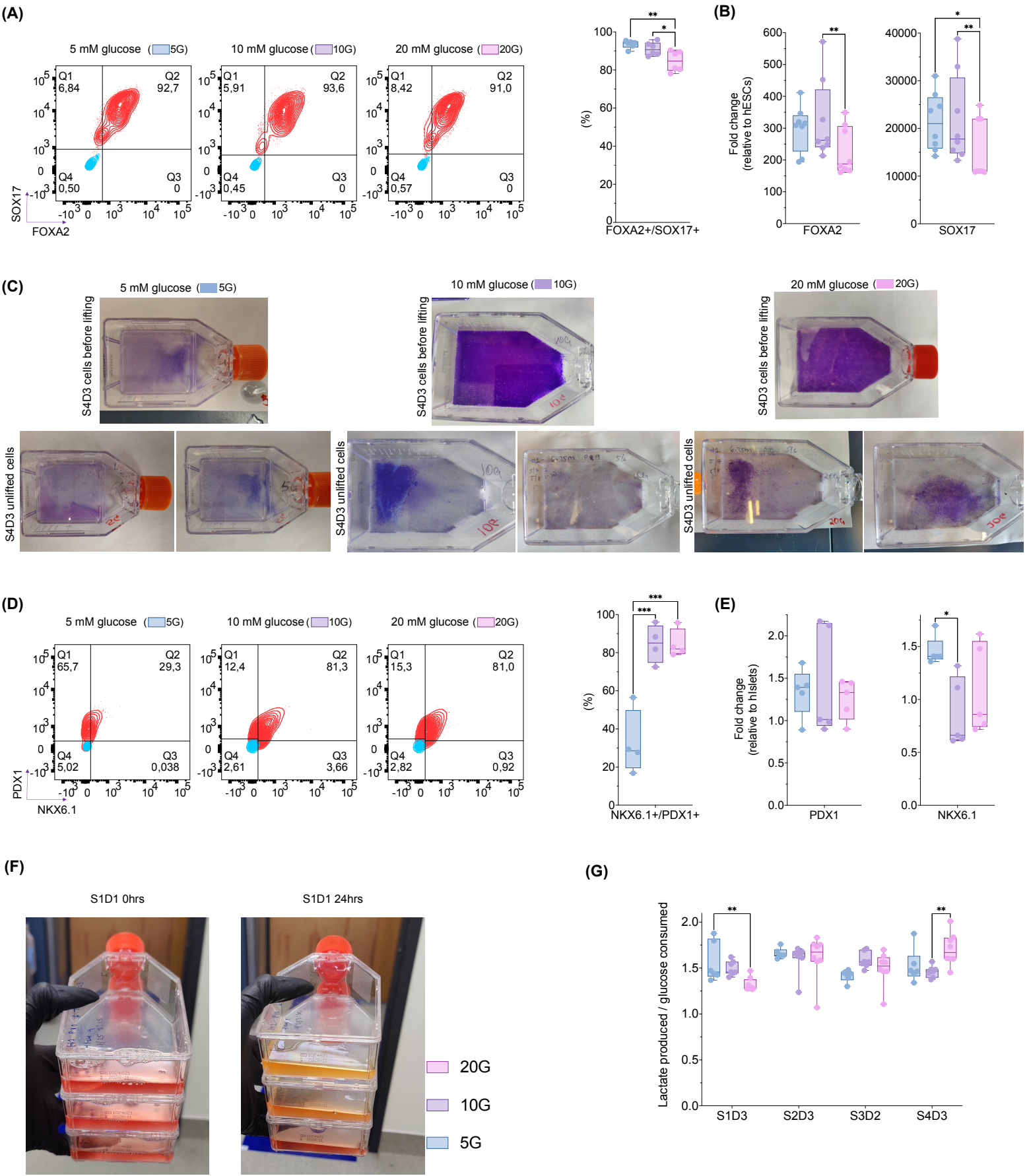

Supplement: Supplementary file 4 — Additional file 4. Figure S4: Effect of glucose concentration on stage-specific markers. (A) Flow cytometry quantification of SOX17+/FOXA2+ at S1D3 with representative flow cytometry plots (n= 6 biological replicates; one-way ANOVA with Tukey post-test). (B) Gene expression profile of SOX17 and FOXA2 at S1D3 (n= 6-8 biological replicates; Friedman test with Dunn’s post-test). (C) Representative crystal violet staining before and after S4D3 lifting. (D) Flow cytometry quantification of NKX6.1+/PDX1+ at S4D3 with representative flow cytometry plots (n= 4 biological replicates; one-way ANOVA with Tukey post-test). (E) Gene expression profile of PDX1 and NKX6.1 at S4D3 (n= 5 biological replicates; Friedman test with Dunn’s post-test). (F) Representative change in phenol red pH indicator during S1D1. (G) Lactate-to-glucose ratio from cells (n = 6-8 biological replicates; two-way ANOVA with Tukey post-test). All box and whisker plots show individual points with median and interquartile range. *P< 0.05, **P< 0.01, ***P< 0.001, ****P< 0.0001. S1D3 = stage 1 day 3, S2D3 = stage 2 day 3, S3D2 = stage 3 day 2, S4D3 = stage 4 day 3 [file 13287_2023_3574_MOESM4_ESM.pdf]

# Supplemental Figure 5

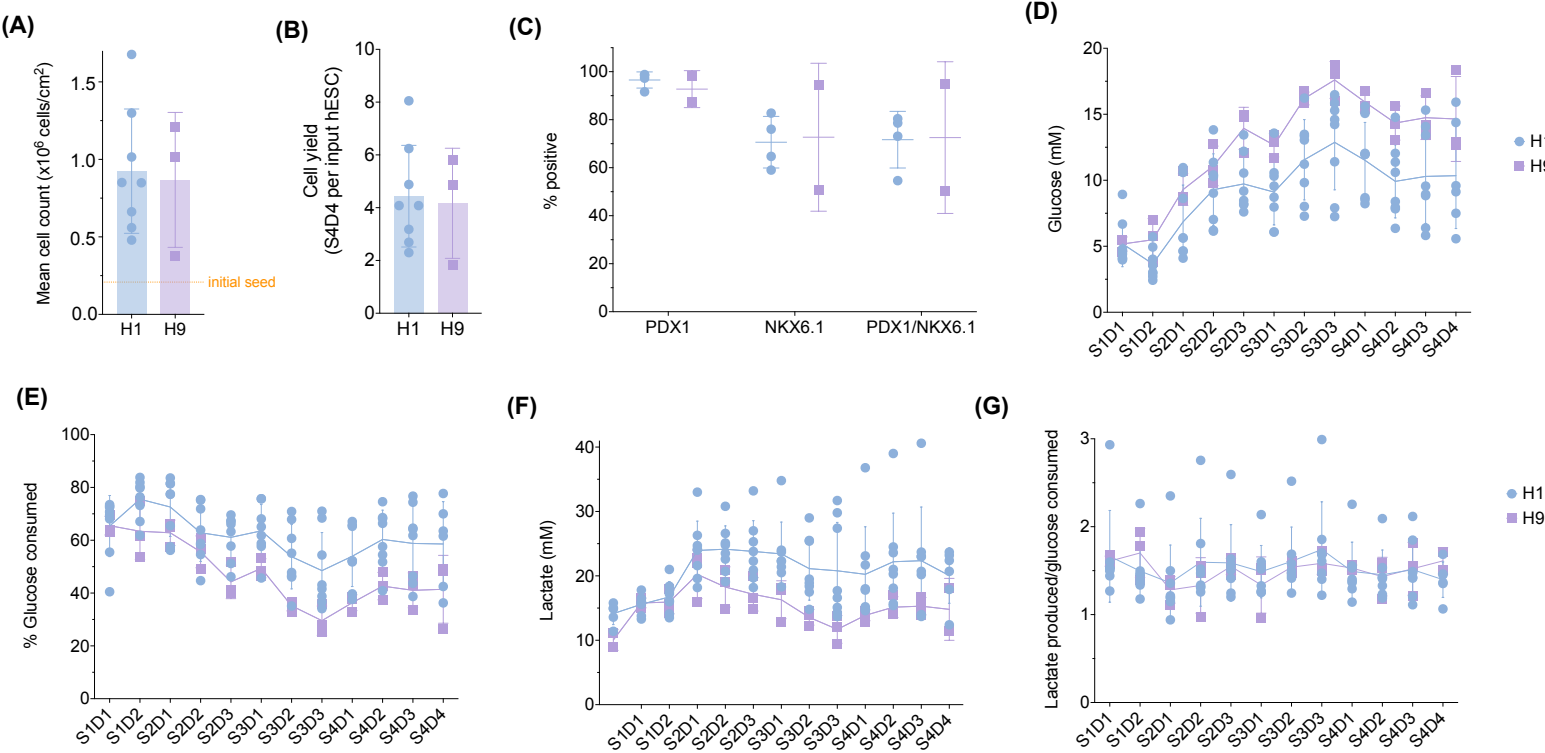

Supplement: Supplementary file 5 — Additional file 5. Figure S5: Nutrient utilization of kit-derived pancreatic progenitors. (A) Cell density of H1 and H9 derived S4D4 cells (orange dash line= initial seeding density). (B) S4D4 cell yield per input H1 and H9 cell. (C) Flow cytometry quantification of NKX6.1+ and PDX1+ positive cells at S4D4 (n=7 for H1 derivatives-12 biological replicates). (D) Glucose remaining in spent media, (E) percentage glucose consumed and (F) lactate concentration measured in spent media between S1D1 and S4D4. (G) Lactate produced per glucose consumed between S1D1 and S4D4 (D-H: n= 7 biological replicates for H1 and n= 3 biological replicates for H9). For panels D-H, the x-axis labels denote the stage and day, e.g. S1D1= stage 1 day 1. Spent media were sampled 24 ± 2 h from the previous media change [file 13287_2023_3574_MOESM5_ESM.pdf]

# Supplemental Figure 6

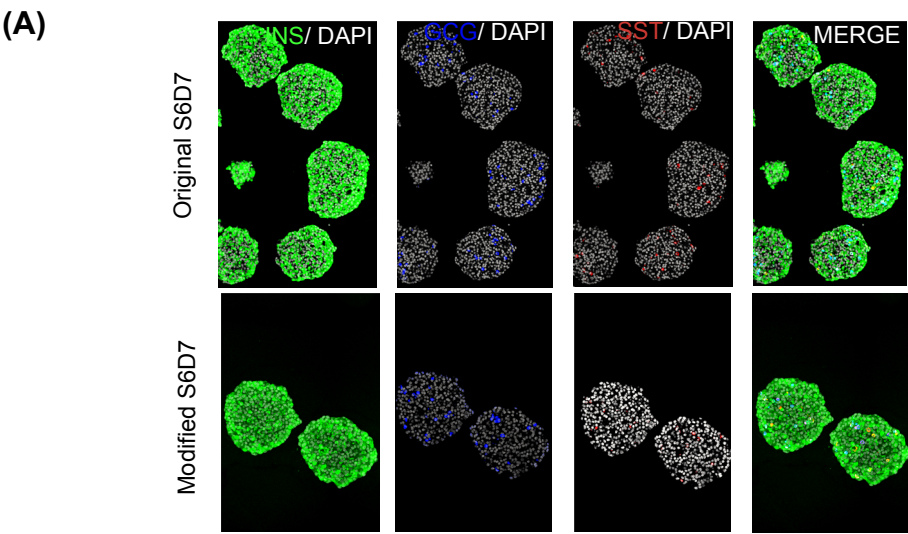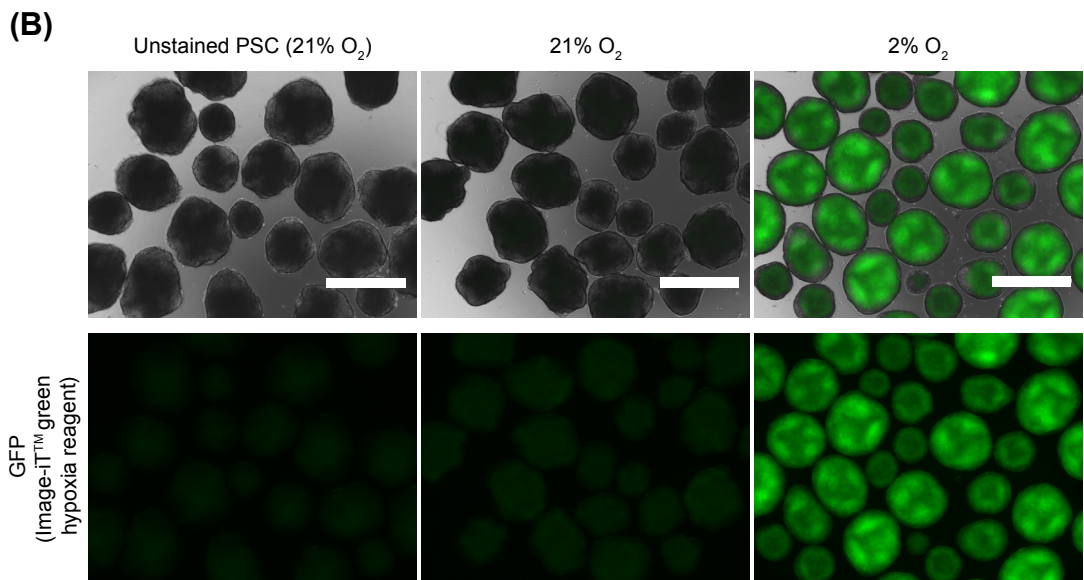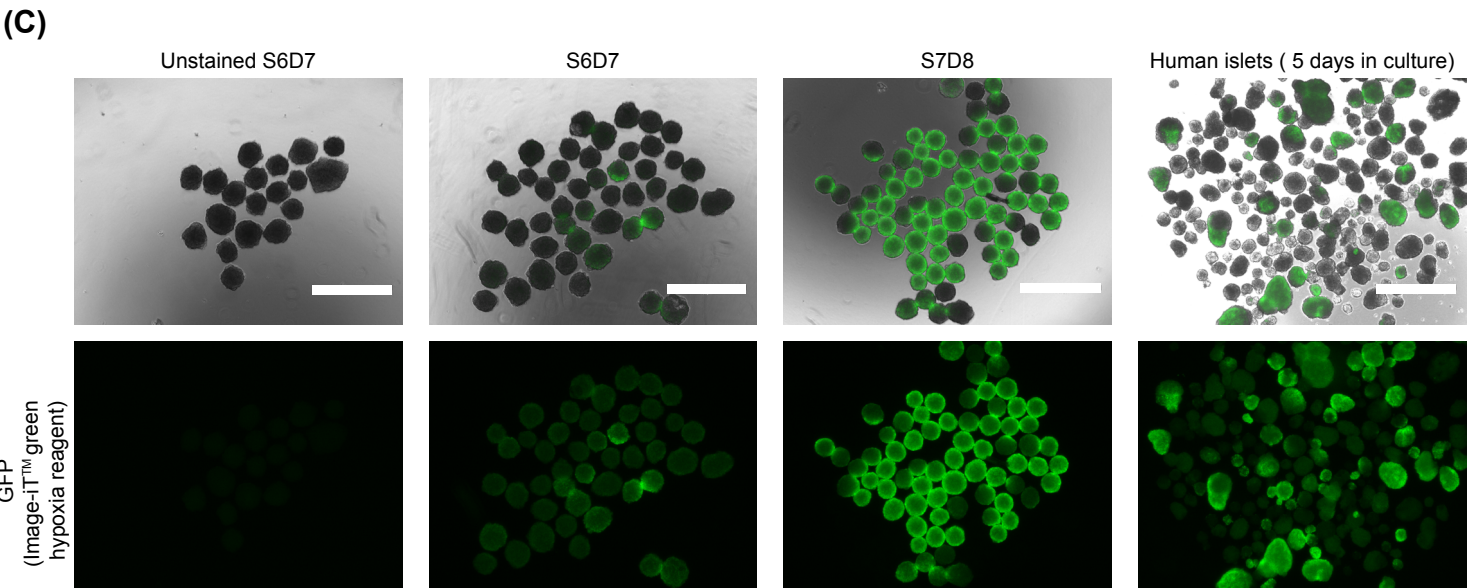

Supplement: Supplementary file 6 — Additional file 6. Figure S6: Staining of later-stage clusters. (A) Representative INS, GCG, SST immunohistochemistry staining of S6D7 clusters generated with the original or modified protocol (B) Hypoxia dye validation using undifferentiated H1 aggregates, scale bar= 750 µm. (C) Hypoxia staining of S6D7 clusters and human islets, scale bar= 750 µm. S5D3 = stage 5 day 3, S6D7 = stage 6 day 7, S7D4 = stage 7 day 4, S7D8 = stage 7 day 8 [file 13287_2023_3574_MOESM6_ESM.pdf]

# Supplemental Figure 7

(A)

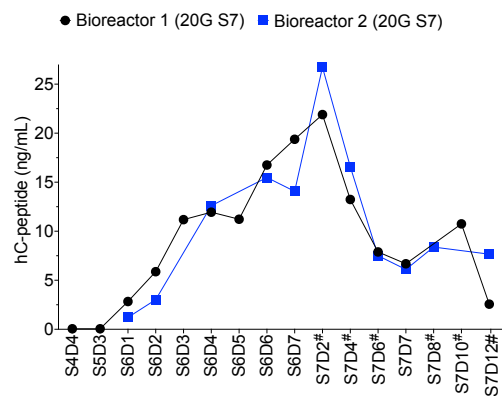

(B)

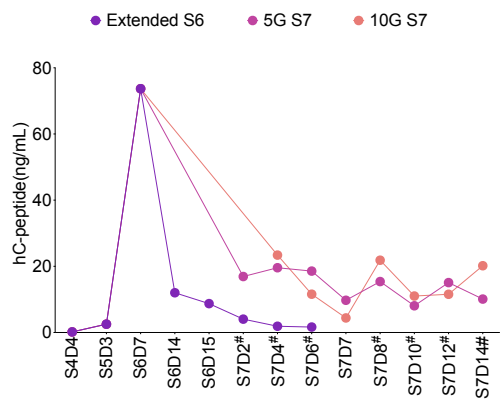

Supplement: Supplementary file 7 — Additional file 7. Figure S7: C-peptide concentration in spent media. (A) C-peptide in spent media with 20 mM glucose (20G) differentiation media during stage 7 (S7). (B) C-peptide in spent media during extended periods of S6 or 5 mM or 10 mM glucose (5G or 10G) during S7. # indicates the spent media were sampled 48 ± 2 h from the previous media change; otherwise, spent media were sampled 24 ± 2 h from the previous media change. The x-axis labels denote the stage and day, e.g., S4D4= stage 4 day 4 [file 13287_2023_3574_MOESM7_ESM.pdf]

# Supplemental Figure 8

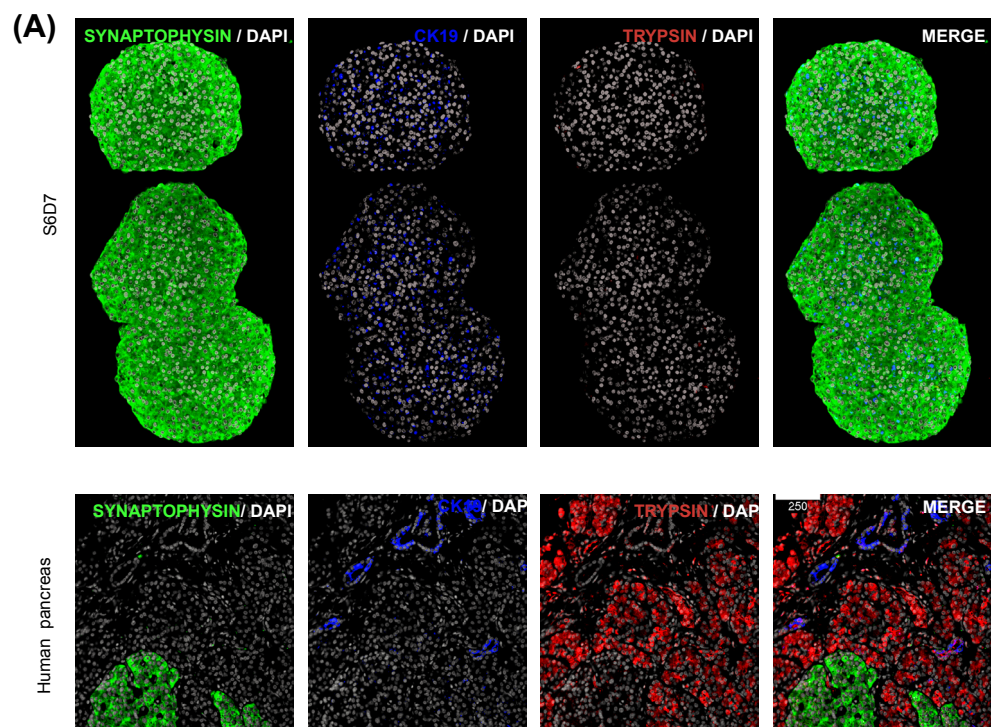

Supplement: Supplementary file 8 — Additional file 8. Figure S8: Immunostaining of clusters. (A) Representative cytokeratin19 (CK19), synaptophysin (SYNP) and trypsin immunohistochemistry staining of S6D7 and human islets [file 13287_2023_3574_MOESM8_ESM.pdf]
